# Extracellular vesicles containing miR-146a-5p secreted by bone marrow mesenchymal cells activate hepatocytic progenitors in regenerating rat livers

**DOI:** 10.1186/s13287-021-02387-6

**Published:** 2021-05-29

**Authors:** Norihisa Ichinohe, Masayuki Ishii, Naoki Tanimizu, Toru Mizuguchi, Yusuke Yoshioka, Takahiro Ochiya, Hiromu Suzuki, Toshihiro Mitaka

**Affiliations:** 1 Department of Tissue Development and Regeneration, Research Institute for Frontier Medicine, Sapporo Medical University School of Medicine, South-1, West-17, Chuo-ku, Sapporo, 060-8556 Japan; 2 Department of Surgery, Surgical Oncology and Science, Sapporo Medical University School of Medicine, Sapporo, Japan; 3 Department of Nursing, Sapporo Medical University School of Health Science, Sapporo, Japan; 4 Division of Molecular and Cellular Medicine, National Cancer Center Research Institute, Tokyo, Japan; 5 Department of Molecular and Cellular Medicine, Institute of Medical Science, Tokyo Medical University, Tokyo, Japan; 6 Department of Molecular Biology, Sapporo Medical University School of Medicine, Sapporo, Japan

**Keywords:** Bone marrow-derived mesenchymal cells, Small hepatocyte-like progenitor cells, Extracellular vesicles, miR146a-5p, Stem cell factor, Interleukin-6

## Abstract

**Background:**

Small hepatocyte-like progenitor cells (SHPCs) appear to form transient clusters in rat livers treated with retrorsine (Ret) and 70% partial hepatectomy (PH). We previously reported that the expansion of SHPCs was amplified in Ret/PH-treated rat livers transplanted with Thy1^+^ cells derived from d-galactosamine-treated injured livers. Extracellular vesicles (EVs) produced by hepatic Thy1^+^ donor cells activated SHPCs via interleukin (IL)-17 receptor B signaling. As bone marrow-derived mesenchymal cells (BM-MCs) also express Thy1, we aimed to determine whether BM-MCs could also promote the growth of SHPCs.

**Methods:**

BM-MCs were isolated from dipeptidyl-peptidase IV (DPPIV)-positive rats. BM-MCs or BM-MC-derived EVs were administered to DPPIV-negative Ret/PH rat livers, and the growth and the characteristics of SHPC clusters were evaluated 14 days post-treatment. miRNA microarrays and cytokine arrays examined soluble factors within EVs. Small hepatocytes (SHs) isolated from an adult rat liver were used to identify factors enhancing hepatocytic progenitor cells growth.

**Results:**

The recipient’s livers were enlarged at 2 weeks post-BM-MC transplantation. The number and the size of SHPCs increased remarkably in livers transplanted with BM-MCs. BM-MC-derived EVs also stimulated SHPC growth. Comprehensive analyses revealed that BM-MC-derived EVs contained *miR-146a-5p*, interleukin-6, and stem cell factor, which could enhance SHs’ proliferation. Administration of EVs derived from the *miR-146a-5p*-transfected BM-MCs to Ret/PH rat livers remarkably enhanced the expansion of SHPCs.

**Conclusions:**

*miR-146a-5p* involved in EVs produced by BM-MCs may play a major role in accelerating liver regeneration by activating the intrinsic hepatocytic progenitor cells.

**Supplementary Information:**

The online version contains supplementary material available at 10.1186/s13287-021-02387-6.

## Background

Fulminant hepatic failure and cirrhosis are severe, and ultimately fatal conditions. Although orthotopic liver transplantation is an effective treatment for these conditions, its use is limited by the scarcity of donor organs and invasiveness to donors [1]. New therapeutic options are needed to enhance regeneration or improve hepatic failure. Recently, regenerative medicine has attracted attention, and cell transplantation may particularly be an alternative to organ transplantation [2, 3].

Liver stem/progenitor cells (LSPCs) and mature hepatocytes (MHs) are considered cellular transplant sources for liver regeneration [3]. Oval cells, small hepatocytes (SHs), and small hepatocyte-like progenitor cells (SHPCs) are well-recognized LSPCs. Oval cells, which are named for their ovoid nuclei, express Thy-1 (CD90), a specific marker of mesenchymal stem cells [4]. Previous reports demonstrated that Thy1^+^ cells emerge transiently in the periportal area soon after d-galactosamine (GalN)-injured liver, and some differentiate into MHs via CD44^+^ hepatocytes [5, 6]. SHs are a subpopulation of MHs that can proliferate clonally and differentiate into hepatocytes in vitro [7, 8]. SHs isolated from adult rat [9] and human livers [10] specifically express CD44, although this expression disappears with maturation [9]. SHPCs have been identified in rat livers treated with retrorsine (Ret) and 70% partial hepatectomy (PH) [11, 12]. Ret is a pyrrolizidine alkaloid that can inhibit hepatocyte division. Although SHPCs emerge 1 week later than PHs and expand to form clusters of small-sized cells, it is difficult to distinguish SHPCs from the surrounding hepatocytes once the liver mass has fully recovered. Recovery of the original liver mass occurs within 10 days in a healthy rat but requires approximately 1 month in a Ret/PH-treated animal [12]. The SHPCs’ origin and their precise location in the liver remain controversial [13–16].

In a previous study, hepatic Thy1^+^ and CD44^+^ cells isolated from GalN-injured livers were transplanted into Ret/PH-treated livers to examine whether LSPCs could repopulate the recipient livers more efficiently than MHs [17]. The number of attached and engrafted hepatic Thy1^+^ cells was very low, and the repopulated cells completely disappeared within two months post-transplantation. CD44^+^ cells were engrafted more efficiently than hepatic Thy1^+^ cells, and some survived longer than one year; however, the repopulation efficiency of these cells was less than that of MHs. Interestingly, hepatic Thy1^+^ cell transplantation induced liver enlargement and accelerated liver regeneration, whereas transplantation with either CD44^+^ cells or MHs did not induce liver enlargement [18]. Further, the numbers and sizes of SHPCs increased in livers transplanted with hepatic Thy1^+^ cells. The administration of extracellular vesicles (EVs) secreted by cultured hepatic Thy1^+^ cells also stimulated liver regeneration and SHPC expansion. These secreted EVs induced interleukin-17 receptor B (IL-17RB), IL-17B, and IL-25 in SHPCs, sinusoidal endothelial cells (SECs), and Kupffer cells respectively. These results indicate that hepatic Thy1^+^ cell transplantation enhances liver regeneration by promoting intrinsic hepatic progenitor cell proliferation via IL17RB signaling.

Transplantation with mesenchymal stem cells (MSCs) can improve liver function in acute hepatic failure and cirrhosis [2, 3]. Moreover, substances secreted by bone marrow-derived MSCs (BM-MCs) are known to exert immunoregulatory and anti-apoptotic effects [19, 20]. Moreover, most BM-MCs express Thy1 [4]. Therefore, we hypothesized that BM-MCs, like hepatic Thy1^+^ cells, might induce the appearance and expansion of SHPCs. In this study, we demonstrated remarkable increases in the numbers and sizes of SHPCs in Ret/PH-treated rat livers that received BM-MC transplantation or were administered BM-MCs-derived EVs. Additionally, we identified *miR-146a-5p*that might play a major role in the growth of LSPCs, which is a different mechanism other than IL-17RB signaling.

## Methods

### Animals

Male F344 rats (dipeptidyl-peptidase IV (DPPIV)^+^ strain; Sankyo Lab Service Corporation, Inc., Tokyo, Japan) and female F344 rats (DPPIV^−^ strain; Charles River Japan, Yokohama, Japan) were used in the present experiment. All animals received proper care, and the Committee of Laboratory Animals approved the experimental protocol following guidelines stipulated by Sapporo Medical University (Approval No.: 17-032, 17-033, 17-034, 19-055, and 20-058).

### BM-MC isolation and culture

According to previously reported methods, rat BM-MCs were obtained from the femurs and tibias of a 5-week-old male F344 rat [21, 22]. The bone marrow was suspended in 1 mL of Dulbecco’s modified Eagle’s medium (DMEM) and broken into a small piece of cells using a syringe with 18-gauge needle (NIPRO, Tokyo, Japan). The cells were dispersed in the control medium [DMEM supplemented with 10% (vol) FBS (MP Biomedicals, Irvine, USA) and antibiotic (1% penicillin and streptomycin solution, Sigma-Aldrich, St. Louis, USA)] and seeded into two 75-cm^2^ culture flasks (Corning Inc, Corning, USA). After incubation at 37 °C in a CO_2_-incubator for 3 days, the non-adherent cells were removed. The adherent cells were cultured further and expanded by exchanging the fresh control medium every 3 days. When the cells reached subconfluency, they were detached from the flasks using phosphate-buffered saline (PBS) containing 0.25% (wt) trypsin and 0.8 mM EDTA. Subsequently, 5 × 10^5^ cells suspended in 0.5 mL of PBS were transplanted.

**Fig. 1:**
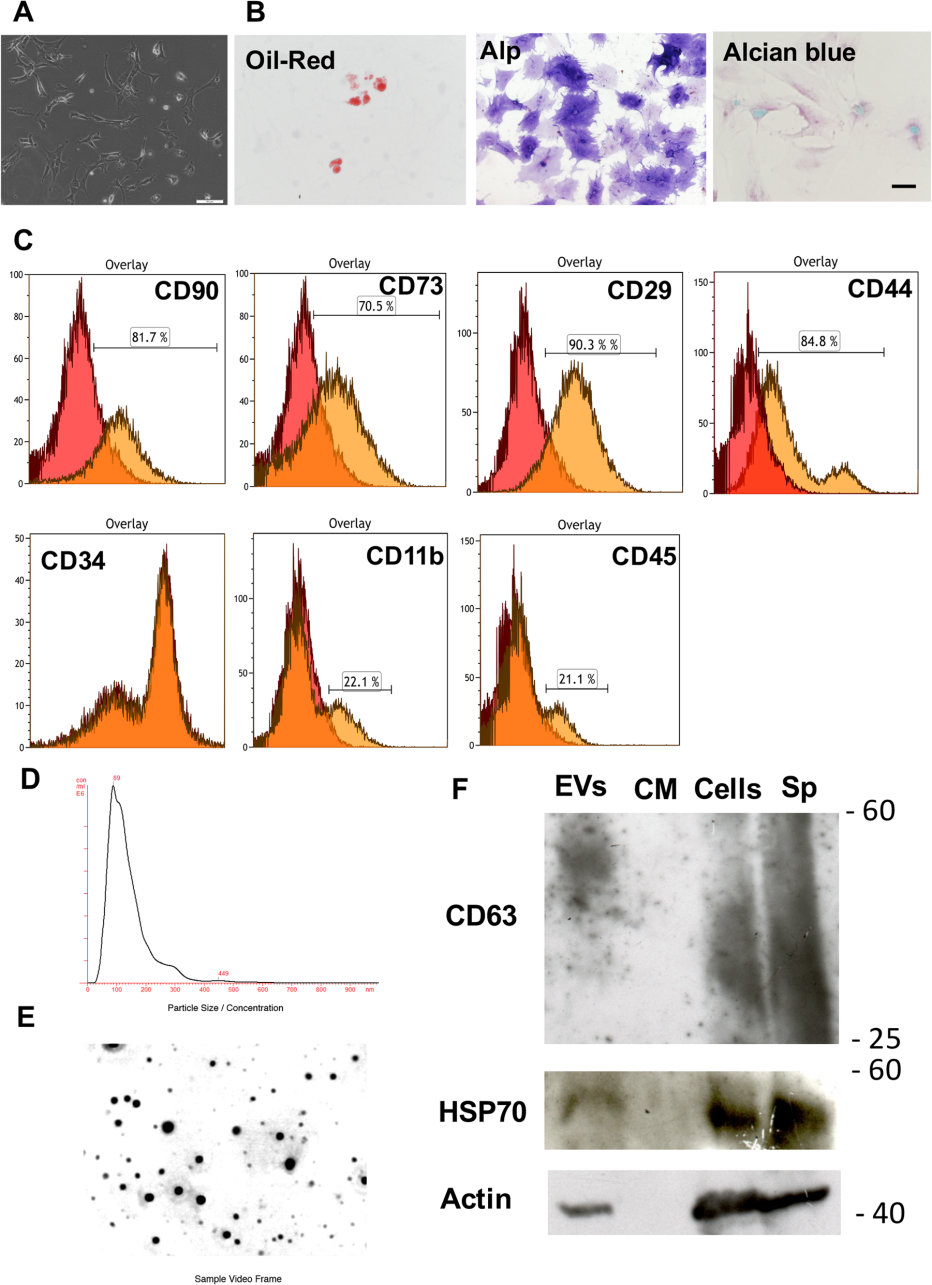
Characterization of BM-MCs and EVs. **a** Phase-contrast image of BM-MCs at day seven after plating. **b** Microscopic images of BM-MCs induced to undergo adipogenesis (Oil-Red; oil red-O staining), osteogenesis (Alp; alkaline phosphatase activity staining), and chondrogenesis (Alcian blue staining). Scale bar, 100 μm. **c** Flow-cytometry analysis of cell surface markers (CD90, CD73, CD29, CD44, CD34, CD11b, CD45) present on BM-MCs. **d** EVs derived from CM of cultured BM-MCs after 48 h were analyzed using the NanoSight particle tracking system. Overall size distribution (histograms) and mode (nm). **e** Sample video frame. **f** Western blots show the existence of CD63, HSP70, and actin by EVs, CM (CM-EVs), and cell lysis (BM-MCs). Spleen (SP) is used as positive control

To confirm their ability to differentiate as stem cells into osteogenic, chondrogenic, and adipogenic cells, BM-MCs were seeded in a 12-well plate at a density of 1 × 10^5^ cells/well. To examine whether BM-MCs could differentiate into osteogenic cells, the BM-MCs were cultured in 1 mL of control medium containing 50 mM l-ascorbic acid and 10 mM β-glycerophosphate with or without 100 nM dexamethasone. Seven days later, the cells were stained to detect alkaline phosphatase activity [21, 22]. PromoCell differentiation-induction media (Heidelberg, Germany) was used to determine BM-MCs’ potential to differentiate into adipocytes or chondrocytes. Briefly, we seeded 5 × 10^4^ BM-MCs per well of a type-I collagen-coated 12-well plate and cultured the cells for 1 week. When the cells reached confluency, the medium was switched to adipogenic differentiation medium (C-28016, PromoCell) or chondrogenic differentiation medium (C-28012, PromoCell), and the cells were cultured for 2 weeks. We evaluated the differentiation potential of adipocytes and chondrocytes using red oil staining and alcian blue staining, respectively (Fig. 1b).

### Flow cytometry

BM-MCs were cultured for 2 weeks after plating and collected by trypsinization. They were washed with PBS and centrifuged at 150×*g* for 5 min. The cells were incubated with mouse anti-rat antibodies specific for CD90, CD73, CD29, CD44, CD34, CD11b, and CD45 in DMEM containing 10% FBS for 30 min at 4 °C. The cells were then washed with PBS containing 2% FBS (wash buffer) and centrifuged at 150×*g* for 5 min. The cells were incubated with a rabbit anti-mouse IgG (H+L) antibody conjugated with Alexa Fluor 488 in DMEM containing 10% FBS for 30 min at 4 °C (Supporting TABLE S1). The incubated cells were washed with wash buffer and centrifuged at 150×*g* for 5 min. The pellet was suspended in a wash buffer containing a propidium iodide solution and passed through a 35-μm cell strainer (Falcon, Corning Inc.). The cells were analyzed on a FACSCanto flow cytometer (BD Biosciences, San Jose, USA). All antibodies used in this study were listed in Supporting Table S1. The data were analyzed using Kaluza Flow Cytometry Software version 1.1 (Beckman Coulter, Inc., Brea, USA).

### Isolation of EVs

EVs were separated from culture medium according to a previously reported method (23). BM-MCs that had been cultured for 12 days were washed with PBS, and the medium was replaced with serum-free DMEM/Nutrient Mixture Ham F-12 (DMEM/F12; Sigma-Aldrich Co., St. Louis, MO) for SHs culture, that is supplemented with 20 mM HEPES (Dojindo Laboratories, Kumamoto, Japan), 25 mM NaHCO_3_ (Kanto Chemical Co. Inc., Tokyo, Japan), 30 mg/L l-proline (Sigma-Aldrich Co.), 0.1% bovine serum albumin (BSA; Serological Proteins Inc., Kankakee, USA), 10 mM nicotinamide (Sigma-Aldrich Co.), 1 mM ascorbic acid-2 phosphate (Asc2P; Wako Pure Chem. Osaka, Japan), 10^−7^ M dexamethasone (Dex; Wako Pure Chem.), 0.5 mg/L insulin-transferrin-selenium (ITS-X; GIBCO-BRL Invitrogen, Grand Island, USA), 10 ng/mL epidermal growth factor (EGF; BD Biosciences), and antibiotics. Forty-eight hours later, the conditioned medium (CM) was collected and centrifuged at 2000×*g* for 10 min at 4 °C. The supernatant was filtered through a 0.22-μm filter (Millipore, Billerica, USA) to remove the cellular debris thoroughly. To prepare EVs, the CM was ultracentrifuged at 110,000×*g* for 70 min at 4 °C [23]. The supernatant was used as CM without EVs (CM-EVs) and the precipitate was re-suspended in 200 μl of saline (0.9% NaCl). The concentration of EVs was measured using a Nano-Drop 1000 spectrometer (Thermo Fisher Scientific, Inc., Waltham, USA), and the protein concentration was determined using a BCA assay kit (Thermo Fisher Scientific, Inc.).

### Western blotting

For western blotting, the EV pellets and CM-EVs, and BM-MCs were lysed in 1x RIPA buffer with 0.1% protease inhibitor cocktail (Sigma-Aldrich). Lysates were centrifuged at 4 °C, and protein concentrations measured using a BCA assay kit. Equal amounts of protein per lane (25 μg) were diluted with LDS sample buffer and loaded onto 12% Acrylamide gels. Proteins were electroblotted to polyvinylidene difluoride membranes (Immobilon-P; Millipore). The membranes were blocked with 5% Block-Ace (Dainippon Pharm, Osaka, Japan) in Tween-Tris-buffered saline. Membranes were incubated 3 h at room temperature with anti-CD63, anti-HSP-70, and anti-actin primary antibodies (Supporting Table S1). Anti-rabbit horseradish peroxidase (HRP)-conjugated or anti-goat HRP-conjugated secondary antibodies were applied. Chemiluminescence was developed with a kit (Super Signal West Dura Extended Duration Substrate; Thermo Fisher Scientific, Inc.). X-ray film was exposed to the light of protein bands which were digitally scanned with a digital scanner (Canon, Tokyo, Japan).

### Transplantation of BM-MCs into Ret/PH livers

Before the transplantation experiment, female F344 rats received two intraperitoneal injections of Ret (30 mg/kg body weight; Sigma-Aldrich) separated by a 2-week interval [11, 12]. PH was performed 2 weeks after the second injection. Primary cultured BM-MCs (5 × 10^5^ DPPIV^+^ donor cells) were administered to the recipient liver via the spleen using a low-dead type syringe with a 19-gauge needle.

### Immunohistochemistry

The antibodies used in this experiment are listed in Supporting Table S1. Recipient rats were euthanized at 14 days post-transplantation, and the livers were immediately harvested and sliced on ice. Five-millimeter-thick samples were embedded in Tissue-Tek (Sakura Finetechnical Co., Tokyo, Japan), frozen in isopentane/liquid nitrogen, and kept at − 80 °C until use. Some slices were fixed in 10% paraformaldehyde/buffered PBS. Enzyme- and immuno-histochemistry for DPPIV were performed to identify donor cells [5, 6]. SHPCs in Ret/PH-treated rat livers were identified as clusters comprising > 10 small-sized hepatocytes, and the sizes were measured using cellSens dimension software (OLYMPUS Corp, Tokyo, Japan).

### Laser microdissection and gene expression analysis

Clusters of SHPCs in the recipient’s livers were collected by using laser microdissection (LMD) following the manufacturer’s protocol [18]. Total RNA was isolated from the captured cells using the RNeasy Mini Kit (Qiagen, Hilden, Germany). SHPCs’ clusters in the recipient livers were collected using LMD according to following the manufacturer’s protocol. Briefly, 7-μm-thick frozen sections were prepared from liver tissues and stained with hematoxylin. The clusters of SHPCs were cut under microscopic observation using an ultraviolet laser (MMI CellCut; Molecular Machines & Industries, Glattbrugg, Switzerland). The gene expression patterns in the cells were analyzed using an oligo microarray spotted with 30,584 probes (SurePrint G3 Rat Gene Expression v2 G4853B, Agilent Technologies, Santa Clara, USA). All DNA microarray data are registered in the GEO database (Accession No. GSE154022).

### Quantitative real-time polymerase chain reaction (qRT-PCR)

For quantitative real-time RT-PCR (qRT-PCR), RNA was reverse-transcribed using an OmniScript RT Kit (Qiagen) and random hexamers as primers. The qRT-PCR analyses were completed using TaqMan RNA sequence-specific probes and Premix Ex Taq (Takara, Shiga, Japan). All qRT-PCRs were performed in triplicate in 96-well optical plates for all samples using an ABI Prism 7500 cycler (Applied Biosystems, Foster City, USA). The relative expression of each gene was normalized to the expression of *Gapdh* as a control. The primers used are listed in Supporting Table S2.

### Gadolinium chloride (GdCl_3_)-induced inhibition of Kupffer cells in Ret/PH models

GdCl_3_ (10 mg/kg; Nacalai Tesque Co, Ltd., Kyoto, Japan) was intraperitoneally administered to Ret-treated rats 24 h before PH as previously reported [18].

### Transplantation of EVs into Ret/PH livers

BM-MCs were cultured for 14 days, and CM was collected as described above. After that, the cells were harvested, and the number of cells was counted. The quantity of EVs was analyzed, and the amount of EVs secreted by 5 × 10^5^ donor BM-MCs was measured. The precipitate by ultracentrifugation of CM was re-suspended in 200 μl of saline and administered to the recipient’s liver via the spleen using a low-dead type syringe with 21-gauge needle (NIPRO, Tokyo, Japan).

### miRNA extraction, miRNA microarray, and qRT-PCR

MicroRNAs (miRNAs) were extracted from EVs using the Qiazol lysis reagent and miRNeasy mini kit (Qiagen). A comprehensive analysis of miRNA expression was performed using a 3D-Gene miRNA Labeling kit and a 3D-Gene miRNA Oligo Chip (Toray Industries, Inc. Tokyo, Japan), which was designed to detect 727 miRNA sequences registered in miRBase release 20.

For the miRNA expression analysis, miRNAs were transcribed into cDNA using the TaqMan MicroRNA Reverse Transcription kit (Applied Biosystems) and the RT primers provided with TaqMan miRNA assays (Applied Biosystems, cat No. 4427975). The cDNA products were analyzed using TaqMan miRNA sequence-specific probes for *U6 small nuclear 2 (U6)*, *miR-144-5p*, *miR-146a-5p*, *miR-146b-5p*, *miR-221-3p*, and *miR-222-3p*, Premix Ex Taq (Takara), and the ABI Prism 7500 sequence detection system (Applied Biosystems) [24]. The primers used are listed in Supporting Table S2.

### Isolation, culture, and passage of small hepatocytes

Primary rat SHs were isolated using the collagenase perfusion method as previously described [25]. SHs were sub-cultured on Matrigel-coated 12-well plates as previously reported [26].

### Coculture with SHs and BM-MCs

Transwell 6-well culture plates (Corning) in which two chambers were separated by a semipermeable (nominal pore size 0.4 μm) membrane were used in indirect cocultures; 5 × 10^4^ BM-MCs and 1 × 10^5^ SHs were plated in the upper and the lower chamber coated with hyaluronic acid, respectively.

### Transfection of mimics to cultured small hepatocytes (SHs)

SHs were sub-cultured at 5 × 10^4^ cells/well on Matrigel-coated 12-well plates and transfected with TaqMan miRNA mimics corresponding to *miR-146a-5p* (Applied Biosystems, MC10722), *miR-146b-5p* (MC13602), *miR-221-3p* (MC10337), *miR-222-3p* (MC11376), and negative controls (Cat No. 4464058) (final concentration: 50 nM). The transfections were performed using Lipofectamine RNAiMAX (Invitrogen, Carlsbad, USA) following the manufacturer’s instructions. Two days later, the medium was replaced, and the SHs were cultured for an additional 5 days, after which the colony growth activity was evaluated.

### Cytokine content analysis

Isolated EVs were lysed in RIPA buffer containing 1 mM protease inhibitors (Sigma-Aldrich). The resulting lysates were assayed to detect BM-MCs-derived proteins using a custom-designed Quantibody rat-specific protein array (RayBiotech, Peachtree Corners, USA, cat. No. QAR-CAA-67) at Cosmo Bio, Ltd. (Tokyo, Japan).

### Morphological analyses of cultured cells

The cultured cells were photographed using a phase-contrast microscope equipped with a CCD camera (Olympus Corp) to count the colonies and cells per colony. Ten fields per dish or well were selected randomly, and three dishes or wells were examined per experiment. At least two independent experiments were performed. All captured images were analyzed using cellSens dimension software (OLYMPUS Corp.).

### Measurement of the labeling index (LI)

Cultured cells were treated with 40 μM 5-bromo-2′-deoxyuridine (BrdU) for 18 h before fixation. The cells were fixed with absolute cold ethanol for 15 min. Subsequently, they were incubated with 2 N HCl for 30 min at RT and then incubated with 0.6% hydrogen peroxide in absolute methanol for 30 min at RT. After blocking with BlockAce for 30 min at RT, the cells were incubated with a mouse anti-BrdU antibody for 60 min. The dishes were rinsed with PBS and subsequently incubated with a biotinylated anti-mouse antibody (Vector Laboratories, Burlingame, USA) for 30 min at RT. Next, the cells were incubated with an avidin-biotin complex solution (VECTASTAIN ABC kit; Vector Laboratories) and treated with 3,3-diaminobenzidine for color development. The number of cells with BrdU-positive nuclei was counted to determine the LI.

### Overexpression of miR-146a-5p in EVs derived from BM-MCs using lentivirus

The transfections were performed using the XMIRXpress vector (SBI System Biosciences, Palo Alto, USA) according to the manufacturer’s instructions [27]. Briefly, 293 TN cells (3 × 10^6^ cells) were plated on the 75-cm^2^ culture flasks. Two μg of transfer plasmid [miR-146a-5p or non-target miRNA (NT)] and 20 μl pPACKH1-plasmid were mixed with 800 μl of serum-free DMEM in tubes and mix by pipetting. Next, 24 μl of PureFection reagent (SBI System Biosciences) were added to tubes, vigorously stirred by a voltex mixer, and incubated at room temperature for 15 min. The mixtures were drop-wised to the flask and swirled to disperse evenly throughout. After 2 days, the media were collected into 12-mL tubes and centrifuged at 3000×*g* for 15 min to pellet cell debris. The viral supernatant was added to the medium of BM-MCs culture. EVs produced by transfected BM-MCs were isolated and evaluated for the expression of miR-146a-5p by qRT-PCR and their abilities to stimulate the growth of SHPCs in Ret/PH models rats as previously described.

### Statistical analysis

The array data were analyzed using Multiple Experiment Viewer software. Microarray data were analyzed using Student’s *t* test, while all other data were analyzed using Tukey’s multiple comparison test. Statistical analyses were performed using GraphPad Prism software (GraphPad Software, La Jolla, USA). Statistical significance was accepted at a probability (p) level < 0.05. The experimental results are expressed as mean ± standard error (SE).

## Results

### Characterization of obtained BM-MCs

After seeding, the BM-MCs immediately adhered to the culture dishes, proliferated, and exhibited an elongated shape, whereas hematopoietic cells floated (Fig. 1a). The cells were cultured in a specific medium for each lineage and underwent cytochemistry for identification to determine whether these cells possessed the capacity to differentiate to adipocytes, osteoblasts, and/or chondrocytes. Some cells differentiated into oil red-O-positive adipocytes and alcian blue-positive chondrocytes, while many cells could differentiate into alkaline phosphatase-positive osteoblasts (Fig. 1b). When the cells reached subconfluency, they were isolated for flow cytometry. We examined the expression of the known MSC positive markers such as CD73, CD90, CD105, CD29, and CD44 [28, 29], in the cell membranes. As shown in Fig. 1c, 81.7%, 70.5%, 90.3%, and 84.8 % of cultured BM-MCs expressed CD90, CD73, CD29, and CD44, respectively, whereas few expressed CD105 (data not shown). The data suggest that, although a majority of BM-MCs are MSCs, about 10–20% of BM-MCs used in this study express the hematopoietic cell markers, CD11b, and CD45 (Fig. 1c). Therefore, the cells used in this study are named BM-MCs, not MSCs, because they are not purified.

### Characterization of obtained EVs

CM from BM-MCs that had been cultured for 48 h was ultracentrifuged to separate EVs and CM-EVs. As shown in Fig. 1d, e, the average particle size was 156 nm. The EVs derived from 5 × 10^5^ BM-MCs contained approximately 6.1 × 10^8^ particles, including 14.0 μg of protein. Western blotting analysis revealed that EVs produced by cultured BM-MCs possessed CD63, HSP70, and Actin (Fig. 1f). We certified that our preparations used in this study composed of mainly EVs derived from BM-MCs.

### Liver histology after BM-MC transplantation

**Fig. 2:**
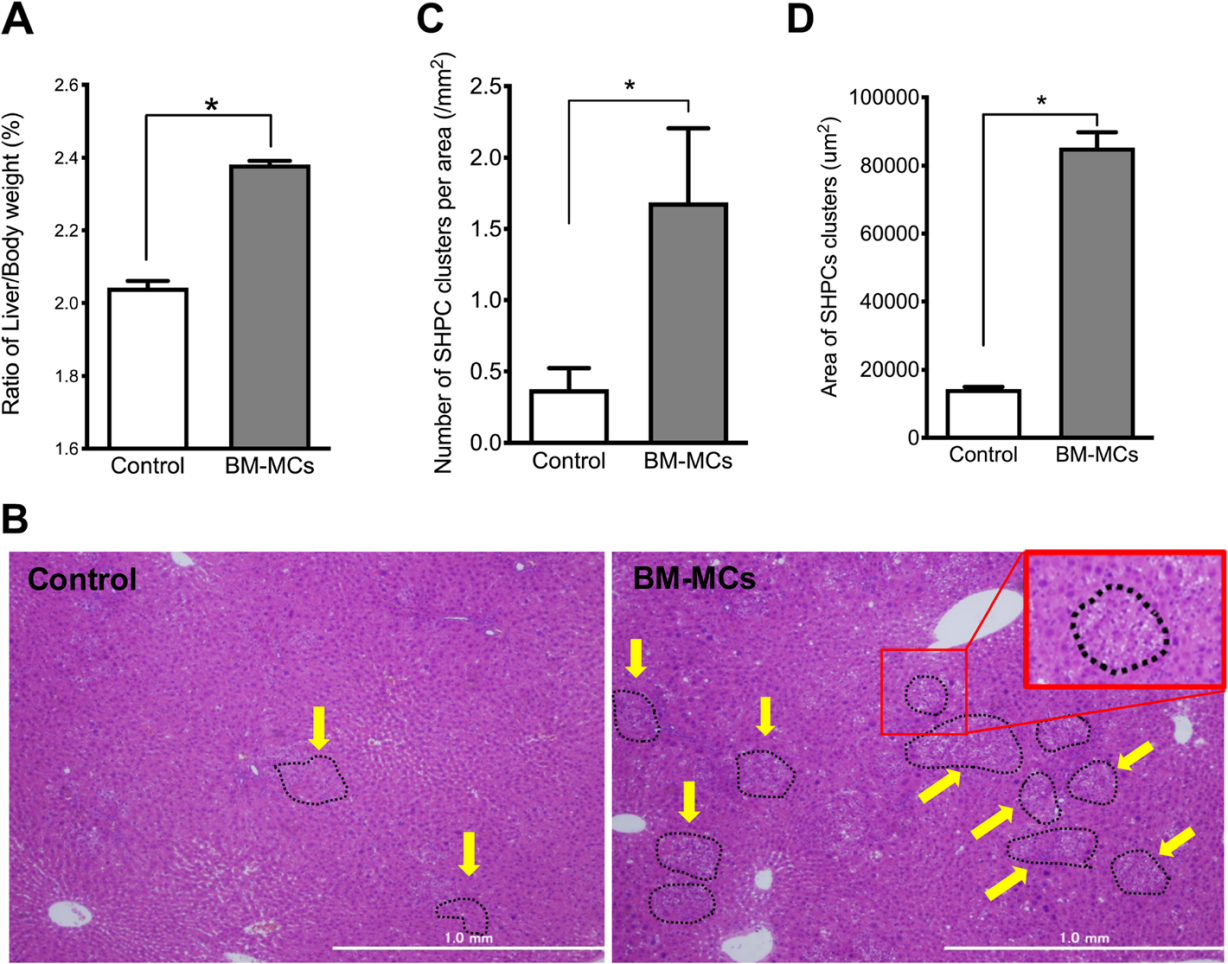
Transplantation of BM-MCs to Ret/PH models. **a** Liver and body weights of rats transplanted with BM-MCs were measured at 14 days after PH. Percentages of liver/body weight were shown. Bars are SEs. An asterisk indicates statistically significant differences, *p* < 0.05. **b** Photos of SHPCs clusters (yellow arrows) in hematoxylin-eosin (H-E) staining of control (left) and BM-MCs transplantation (right). Inset in the right side photo is an enlarged typical SHPCs (red square) shown in the livers with BM-MCs transplantation. Fine fat droplets (empty vesicles) are observed in the cytoplasm of SHPCs. Scale bars, 1 mm. The number of SHPC clusters per area (**c**) and area of the cluster (**d**) were measured 14 days after transplantation. Asterisks indicate statistically significant differences, *p* < 0.05

The liver weights of Ret/PH model rats transplanted with BM-MCs had increased remarkably at 14 days post-transplantation (Fig. 2a). The recipient’s livers were analyzed histologically at 14 days post-transplantation to examine the cause of this enlargement. Enzyme-histochemistry to detect DPPIV^+^ donor cells did not identify these cells in the recipient livers (data not shown). As shown in Fig. 2b, however, many SHPC clusters comprising small-sized cells were observed in the livers transplanted with BM-MCs, and most of these cells had accumulated fine fat droplets in the cytoplasm (Fig. 2b, inset). The distribution of SHPC clusters was not limited to the defined area of a liver lobule. The number and average area of SHPC clusters in livers transplanted with BM-MCs were four and six times greater than those in a non-transplanted liver (Control), respectively (Fig. 2c, d). In other words, the enlargement of BM-MC-transplanted livers may be due to an expansion of SHPCs.

### Characterization of SHPCs in livers transplanted with BM-MCs

**Fig. 3:**
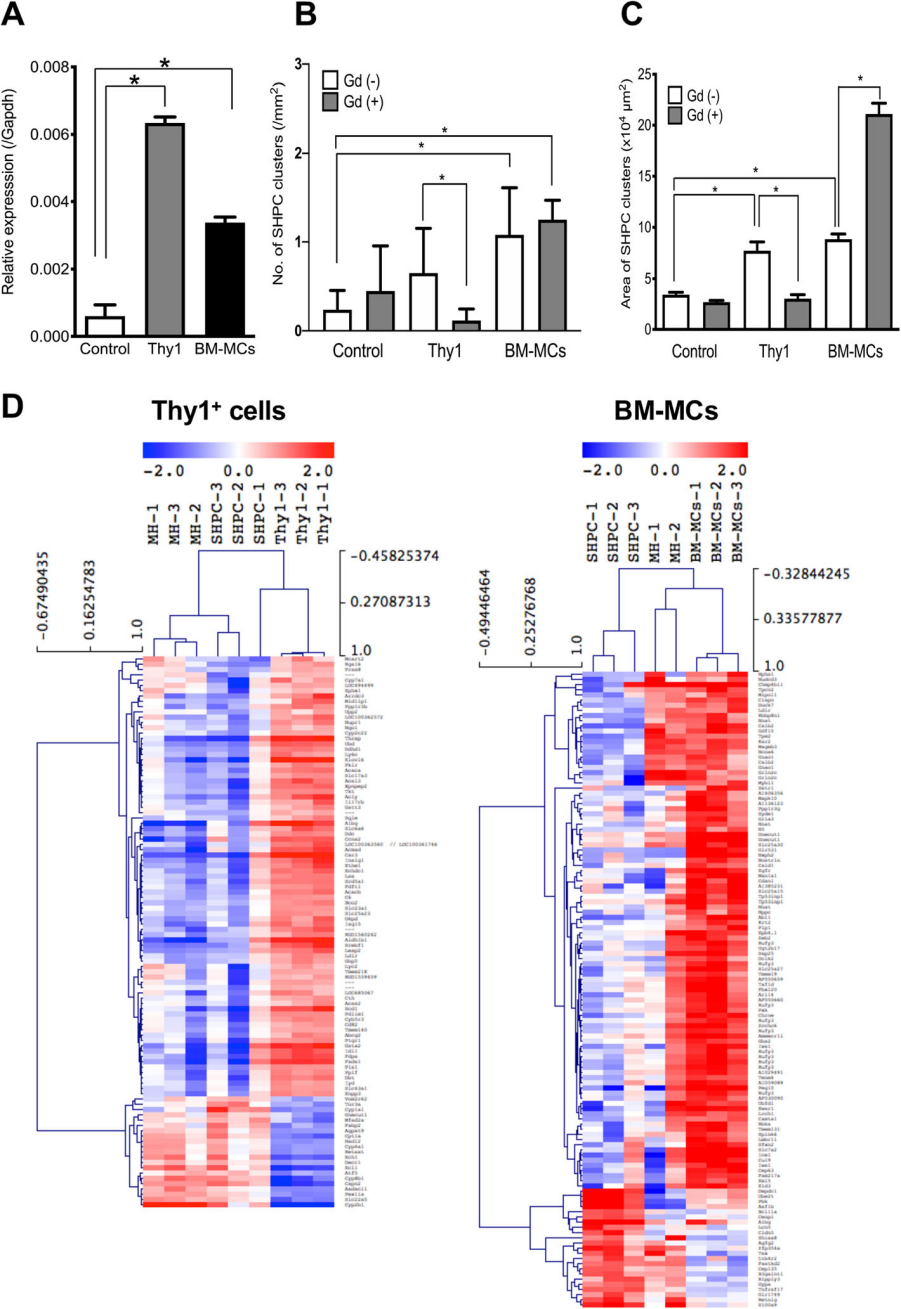
Effects of Gadlium treatment on the SHPC behavior, and characterization of SHPCs in livers transplanted with BM-MCs. **a** *IL17rb* gene expression in SHPCs of livers receiving Thy1^+^ cells and BM-MCs transplantation was confirmed by qRT-PCR. Kupffer cells in Ret/PH-treated rat livers were inactivated by administering GdCl_3_ 24 h before cell transplantation. The number of SHPC clusters per liver area (**b**) and the size of SHPC clusters (**c**) were measured using liver samples of 14 days after cell transplantation. Bars show SEs. Asterisks indicate statistically significant differences; *p* < 0.05. **d** Clustering analysis of the gene profiles of SHPCs with Thy1^+^ cells and BM-MCs. Significantly upregulated genes were analyzed using clustering methods in Thy1^+^ cells and BM-MCs

As previously reported [18], EVs secreted by donor cells stimulated SHPC growth via IL17RB signaling in the livers transplanted with hepatic Thy1^+^ cells. Therefore, we first verified whether transplanted BM-MCs could activate SHPC growth similarly. Because transplantation with hepatic Thy1^+^ cells induced the expression of IL-17RB, IL17-B, and IL-25 in SHPCs, SECs, and Kupffer cells, respectively, we subjected BM-MC-transplanted and non-transplanted livers to double immunohistochemistry for IL-17RB/HNF-4α, IL-17B/SE-1, and IL25/CD68. As shown in Supplemental Figure 1, IL-17RB expression was increased in the SHPCs from BM-MC-transplanted livers. Although CD68^+^ Kupffer cells expressed IL-25, SE1^+^ SECs did not clearly express IL-17B. Cell clusters in livers transplanted with hepatic Thy1^+^ cells or BM-MCs were separated using LMD (Supplemental Figure 2A) to confirm the expression of *Il17rb* in SHPCs. Although *Il17rb* expression was upregulated more strongly in SHPCs transplanted with either BM-MCs or hepatic Thy1^+^ cells relative to the control, the expression tended to be higher in SHPCs exposed to hepatic Thy1^+^ cells (Fig. 3a). These results indicate that, although IL-17RB signaling is involved in expanding SHPCs in livers transplanted with BM-MCs, other factors may contribute to this phenomenon.

To clarify the involvement of other mechanisms, we examined whether the administration of GdCl_3_, which can inactivate Kupffer cells, would inhibit the expansion of SHPCs in the livers transplanted with BM-MCs. Consistent with a previous report [18], GdCl_3_ administration reduced the numbers and areas of SHPCs in livers transplanted with hepatic Thy1^+^ cells (Fig. 3b, c). However, in the livers transplanted with BM-MCs, the average area of SHPCs increased remarkably, whereas the number of SHPCs did not increase. Although dormant SHPCs may not have been activated, the suppression of Kupffer cell activity led to the growth of SHPCs. Potentially, mechanisms other than IL-17RB signaling may also contribute to the expansion of SHPCs in the livers transplanted with BM-MCs. The further expansion of SHPCs in response to Kupffer cell phagocytotic activity inhibition may also demonstrate the direct effects of donor cells on neighboring cells during relatively long retention in the sinusoids.

To investigate the other mechanisms that stimulated SHPCs, we examined the characteristics of SHPCs with or without BM-MC transplantation. We performed a comprehensive analysis of gene expression in SHPCs separated by using LMD. As shown in Fig. 3d, the gene expression patterns differed between SHPCs induced by hepatic Thy1^+^ cells and BM-MCs. SHPCs induced by BM-MCs had a gene expression pattern similar to that of MHs around SHPCs than to that of SHPCs induced by hepatic Thy1^+^ cells. Overall, SHPCs induced by BM-MCs tended to be more differentiated than those induced by hepatic Thy1^+^ cells. In fact, the expression of *Cyp1a2* and *Cyp2b1* of SHPCs with BM-MCs were significantly higher than those of controls by qRT-PCR (Supplemental Figure 2B). The fold changes and *p* value of significance were measured in the expression of all genes in SHPCs with or without BM-MCs transplantation, and the results were presented as volcano plots (Supplemental Figure 3A). The number of genes significantly up-regulated was 275, and that of genes down-regulated was 75. The gene list uploaded in the DAVID and KEGG pathway website indicated that the upregulation of Mapk signaling pathway (Supplemental Figure 3B) and downregulation of p53 signaling pathway (Supplemental Figure 3C). The expression of the pattern of the Mapk signaling gene and p53 signaling genes were shown as a heatmap in Supplemental Figures 3D and 3E, respectively. We performed qRT-PCR and confirmed that the expressions of *Mapk1 and Jun* were significantly increased in SHPCs of livers with BM-MCs. However, there were no significant differences in genes of *p53*, *p27*, *p21*, and *p16* concerning p53 signaling in SHPCs with and without BM-MCs transplantation (Supplemental Fig 3F).

### Analysis of soluble factors isolated from the conditioned medium of BM-MCs

**Fig. 4:**
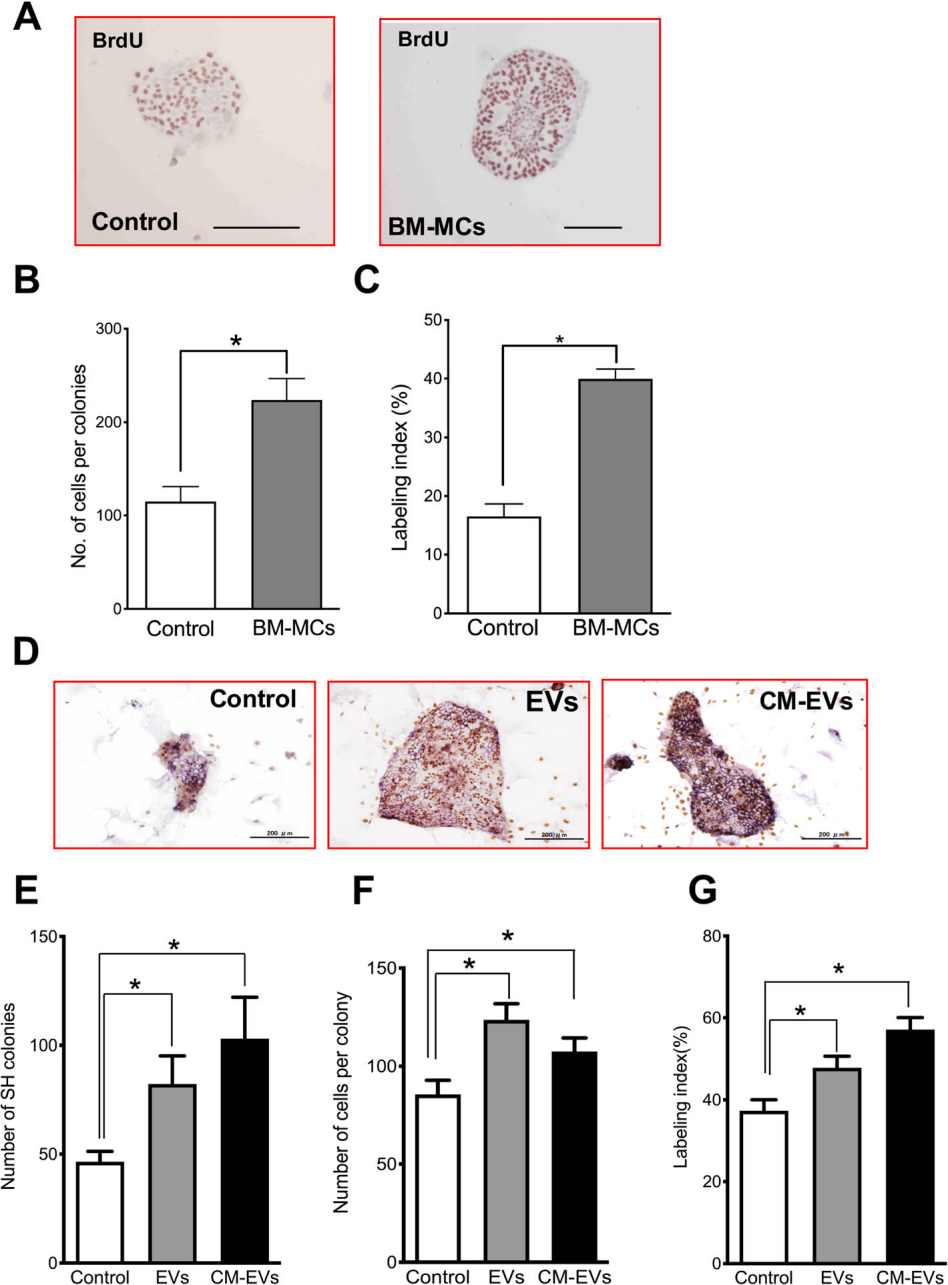
Effects of soluble factors derived from BM-MCs on the colony formation of small hepatocytes. **a** Photos show the immunohistochemistry for BrdU of typical SH colonies at 7 days after plating. SHs were indirectly cocultured with BM-MCs by using a cell strainer. Brown nuclei were BrdU-positive, most of which are mononucleate. Scale bars, 100 μm. **b** The number of cells per colony was measured. **c** The percentage of BrdU^+^ cells per colony is calculated. Bars show SE. Asterisks indicate statistically significant differences; *p* < 0.05. Panel **d** shows the photos of typical SH colonies treated with EVs and CM-EVs derived from BM-MCs at 7 days after the treatment. Immunocytochemistry for BrdU and hematoxylin-staining were performed. Scale bars, 100 μm. The number of SH colonies (**e**), the number of cells per colony (**f**), and the percentage of BrdU^+^ cells per colony (**g**) are measured. Bars show SEs. Asterisks indicate statistically significant differences; *p* < 0.05

After confirming that BM-MCs induced SHPC emergence and growth, we investigated whether soluble factors secreted by BM-MCs could induce hepatic progenitor cell proliferation. BM-MCs were initially cocultured with SHs isolated from a healthy adult rat liver. As shown in Fig. 4a–c, an indirect coculture with BM-MCs enhanced the growth of SH colonies. The number of SHs in a colony cocultured with BM-MCs for 7 days was approximately two times larger than that in the control colony. Also, the labeling index (LIs) of cocultured and Control SHs were approximately 40% and 17%, respectively.

Either 1.4 μg (10 μl) of EVs or 179.1 μg (100 μl) of CM-EVs were added to the SH culture medium at 3 h after plating to identify the soluble factors that could stimulate hepatic progenitor cell growth, and the medium was replaced with fresh medium lacking CM-derived substances after 48 h. As shown in Fig. 4d, the immunocytochemistry analysis of BrdU shows that the addition of either EVs or CM-EVs accelerated the growth of SHs. Seven days after plating, the numbers of SH colonies in cultures treated with EVs or CM-EVs were approximately two and 2.5 times larger, respectively, than that in the control culture (Fig. 4e), and the corresponding sizes of the colonies were approximately 1.5 and 1.3 times larger, respectively (Fig. 4f). More than 50% of SHs exposed to medium with EVs and CM-EVs were BrdU^+^ SHs, compared to approximately 40% of Control SHs (Fig. 4g).

**Fig. 5:**
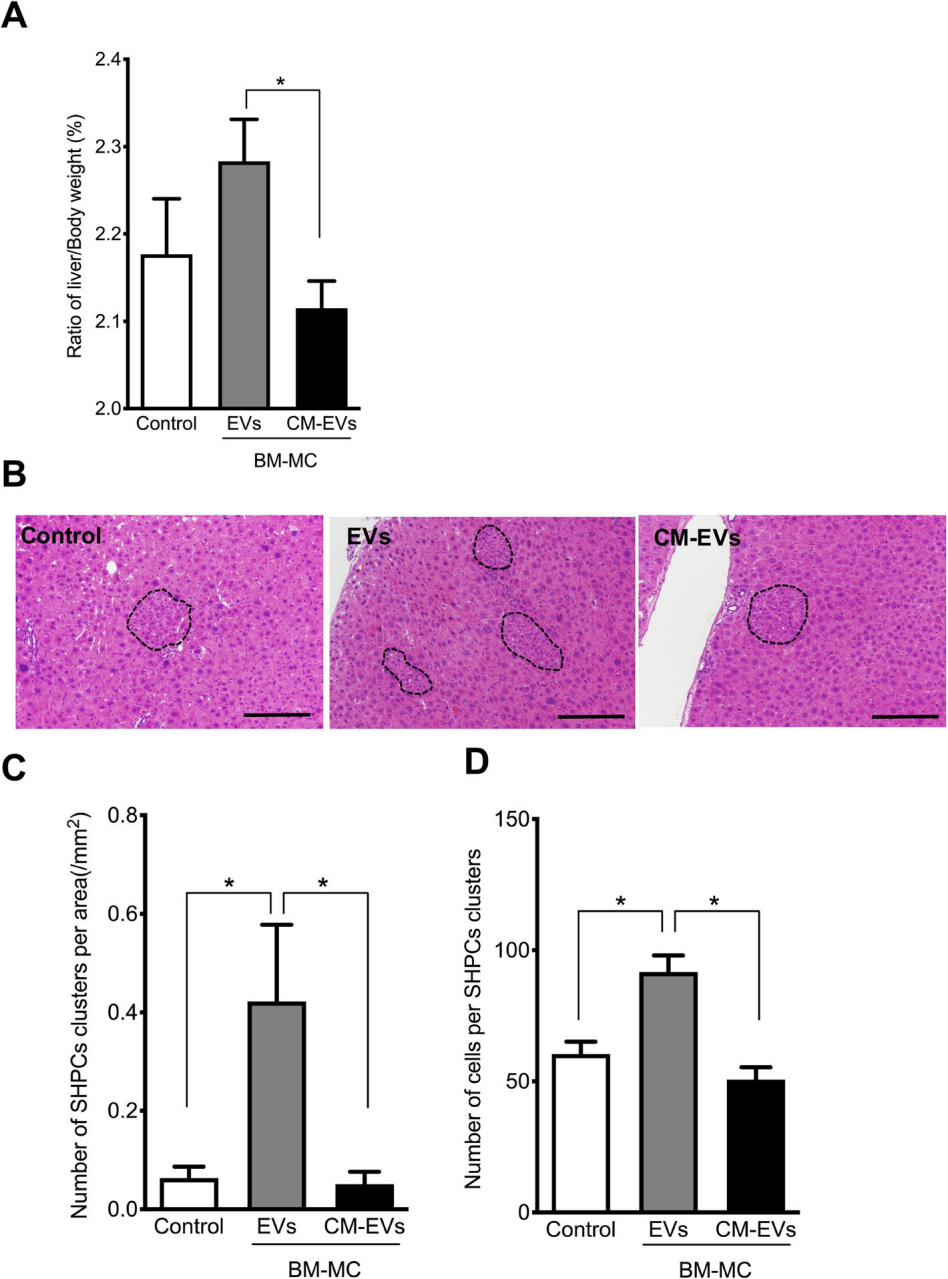
Administration of EVs secreted by cultured BM-MCs to Ret/PH model rat livers. EVs and CM-EVs derived from the conditioned medium of cultured BM-MCs for 48 h were administered via spleen, and the livers were examined at 14 days post-administration. **a** Liver and body weights of rats treated with EVs or CM-EVs were measured 14 days after the treatment. Bars show SE. Asterisks indicate statistically significant differences; *p* < 0.05. **b** Photos of SHPC clusters in the livers of control (left), EVs (center), and CM-EVs (right). The samples were stained with H-E. Scale bar, 200 μm. **c** The number of SHPC clusters per liver area and **d** the number of cells per cluster is shown. Bars show SEs and asterisks indicate statistically significant differences, *p* < 0.05

Next, we examined the effects of EVs and CM-EVs on the growth of SHPCs. The EVs derived from 5 × 10^5^ BM-MCs or CM-EVs were administered to Ret/PH-treated rat livers via the spleen. As shown in Fig. 5a, liver regeneration was more strongly enhanced in the rats treated with EVs than in those with CM-EVs at 14 days post-administration. Histological analysis revealed that EVs stimulated SHPC growth, whereas CM-EVs did not (Fig. 5b). The numbers and sizes of SHPC clusters in the livers with EVs were increased significantly, whereas CM-EVs did not stimulate the growth of SHPCs (Fig. 5c, d).

### Identification of growth-related agents contained in EVs secreted by cells

**Fig. 6:**
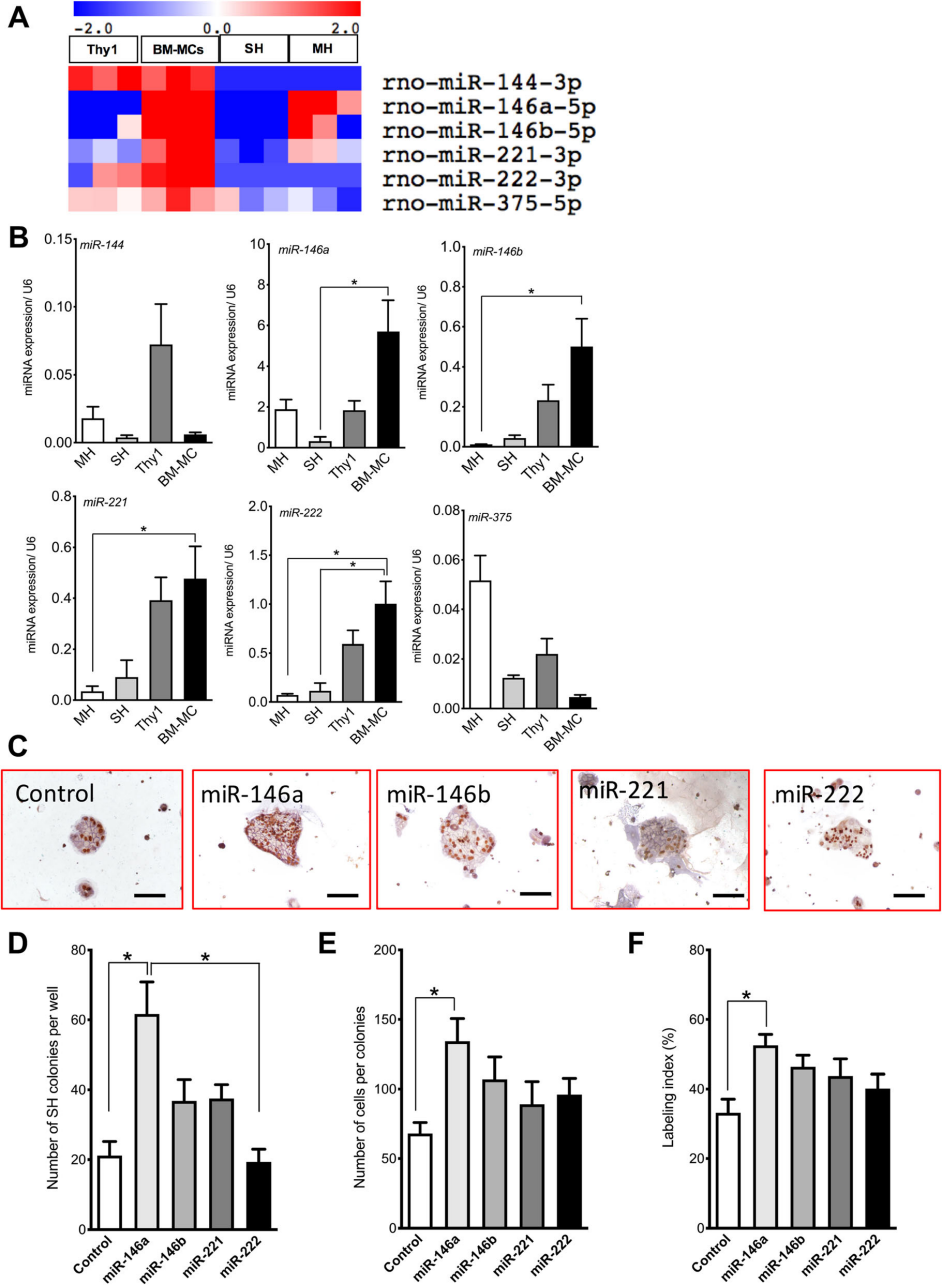
Effects of miRNAs abundantly contained in EVs secreted by BM-MCs on the proliferation of SHs. **a** Six miRNAs up-regulated 5 times higher than those of hepatic Thy1^+^ cells are shown as a heatmap. **b** Amounts of *miR-144-3p, miR-146a-5p, miR-146b-5p, miR-221-3p, miR-222-3p*, and *miR-375-5p* in the EVs secreted by SHs, hepatic Thy1^+^ cells, and BM-MCs were confirmed by using qRT-PCR. Four of six miRNAs were included more abundantly in EVs secreted by BM-MCs than in other cells. Bars show SEs and asterisks indicate statistically significant differences, *p* < 0.05. **c** These photos show the SH colonies, which mimics those 4 miRNAs, *miR-146a-5p, miR-146b-5p, miR-221-3p,* and *miR-222-3p*, were transfected by adding to the culture medium for 2 days. The cells were then cultured until day seven and BrdU was added in culture medium 24 h before fixation. Immunocytochemistry for BrdU was conducted. Scale bars, 100 μm. The total number of SH colonies per well (**d**) and the number of cells per colony (**e**), and the percentage of BrdU^+^ cells per colony (**f**) were measured. Asterisk indicates statistically significant differences compared with control, *p* < 0.05

EVs are enriched with many bioactive molecules, such as proteins, lipids, RNA, and mitochondrial DNA [30]. MSC-derived EVs are reported to contain many miRNA (> 150) and proteins (> 850), including growth factors and cytokines [31]. Therefore, we examined which miRNAs were included in the EVs derived from cultured BM-MCs and hepatic Thy1^+^ cells. We used the miRNA Oligo Chip to perform a comprehensive analysis of miRNAs (Supplemental Figure 4). We then selected miRNAs (*miR-144-3p*, *miR-146a-5p*, *miR-146b-5p*, *miR-221-3p*, *miR-222-3p*, and *miR-325-5p*) that were present at 5 times higher levels in EVs derived from BM-MCs relative to those from hepatic Thy1^+^ cells (Fig. 6a). Then, the expression of those miRNAs was confirmed by qRT-PCR. The expression of *miR-146a-5p*, *miR-146b-5p*, *miR-221-3p*, and *miR-222-3p* was relatively higher in EVs from BM-MCs than in those from Thy1^+^ cells (Fig. 6b). Therefore, these miRNAs were subjected to further analysis.

Mimics of these miRNAs were administered to cultured SHs, and the growth of these cells was evaluated (Fig. 6c). Seven days after treatment, the numbers of colonies per dish and cells per colony had increased significantly in the cells treated with the *miR-146a* mimic (Fig. 6d, e). The ratio of BrdU^+^ cells was also significantly increased in the cells treated with the *miR-146a* mimic (Fig. 6f). These results indicate that a mimic of *miR-146a-5p* could enhance the growth of hepatocytic progenitor cells.

### Identification of growth factors/cytokines contained in EVs secreted by BM-MCs

**Fig. 7:**
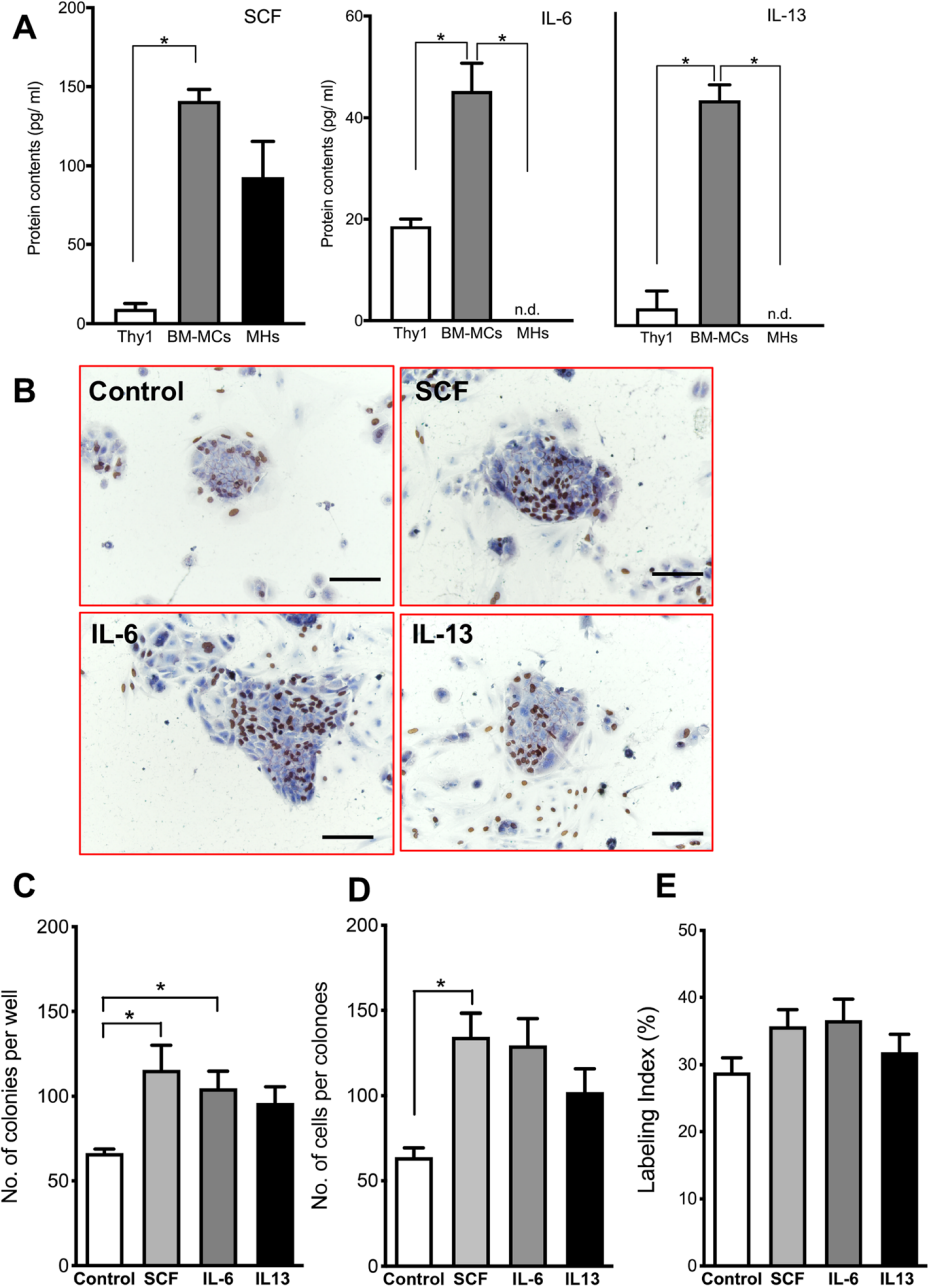
Effects of cytokines included in EVs derived from BM-MCs. **a** The amounts of cytokines included in EVs secreted from hepatic Thy1^+^ cells, BM-MCs and MHs were measured using a custom-designed Quantibody rat-specific protein array (QAR-CAA-67). SCF, IL-6, and IL-13 were included in EVs secreted from BM-MCs much more than ones from Thy1^+^ cells, and IL-6 and IL-13 were included in EVs secreted from BM-MCs much more than ones from MHs. **b** SCF, IL-6, and IL-13 were added to the culture medium 3 h after plating and refreshed every other day. Panel **b** shows the photos of typical SH colonies treated with each cytokine. BrdU was added to the culture medium 24 h before fixation, and immunocytochemistry for BrdU was performed at 7 days after plating. Scale bars, 100 μm. The total number of SH colonies per well (**c**), the number of cells per colony (**d**), and the percentage of cells with BrdU^+^ nucleus per colony (**e**) are shown. Asterisk indicates statistically significant differences, *p* < 0.05

Because EVs were shown to induce SH growth (Fig. 4d–g), we comprehensively evaluated the factors produced by BM-MCs but not by hepatic Thy1^+^ cells (Supplemental Fig 5). We then selected the factors specifically expressed at two-fold higher levels in BM-MCs than in hepatic Thy1^+^ cells. We selected SCF, IL-6, and IL-13 as candidates through a cytokine array (Fig. 7a). To examine whether these factors could stimulate the growth of SHs, we added SCF, IL-6, and IL-13 to the SH culture medium individually and evaluated the cell growth after 7 days (Fig. 7b–e). Although SCF and IL-6 both increased the number of colonies (Fig. 7c), only SCF significantly increased the number of cells per colony (Fig. 7d). However, DNA synthesis in SHs at day seven did not differ significantly between the cells treated with SCF, IL-6, or IL13 (Fig. 7e). These results suggest that SCF and IL6 could plausibly stimulate SH growth.

**Fig. 8:**
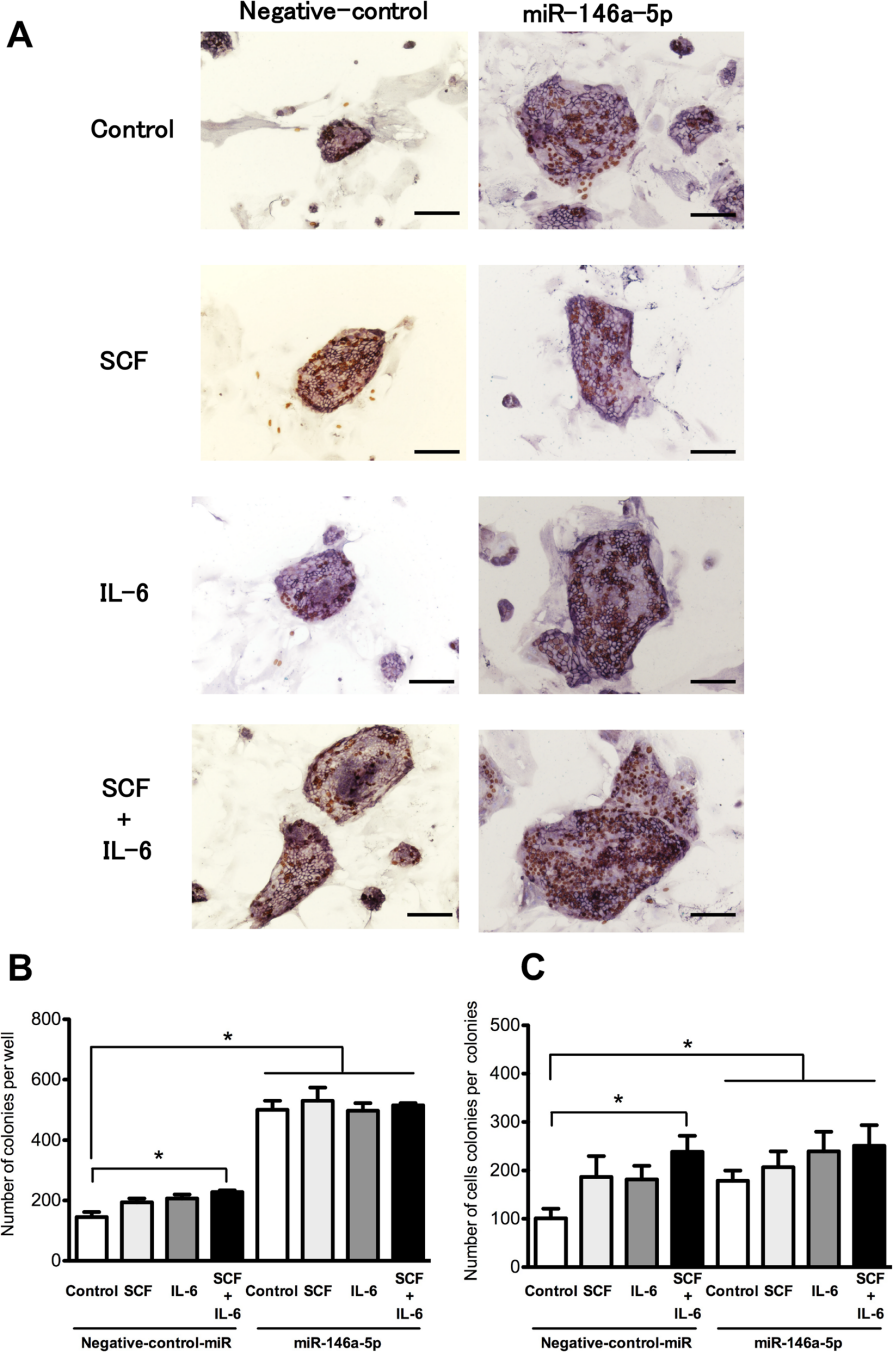
Effects of SCF and IL-6 on the growth of SHs transfected with *miR-146a-5p*. SHs were plated on 12-well plates and cultured for 3 h. After the culture medium was changed, the combinations of *miR-146a-5p* mimic and cytokine were added to the medium and cultured for 48 h. After that, fresh medium without cytokines was replenished every other day. Panel **a** shows the photos of typical SH colonies treated with each cytokine. BrdU was added to the culture medium 24 h before fixation, and immunocytochemistry for BrdU was performed 7 days after plating. Scale bars, 100 μm. The total number of SH colonies per well (**b**) and the number of cells per colony (**c**) were measured. Asterisk indicates statistically significant differences, *p* < 0.05

Next, we examined whether SH growth would be enhanced additively by the combined administration of *miR-146a-5p* and cytokines. IL-6 and SCF were added to the SH culture medium for the initial 48 h (Fig. 8a). As shown in Fig. 8b, c, in cells treated with negative control miRNA, the combination of SCF and IL-6 significantly increased the number and sizes of SH colonies. Furthermore, in cells treated with *miR-146a-5p*, the addition of cytokines led to remarkable increases in the number and sizes of SH colonies relative to the negative control. However, each cytokine did not additively enhance the growth of SHs, suggesting that *miR-146a-5p* may be the most significant factor in the expansion of hepatic stem/progenitor cells.

### Expression of genes encoding growth factor receptors in SHPCs induced by BM-MC transplantation

To confirm whether cytokines might be related to the enlargement of SHPC clusters in livers transplanted with BM-MCs, we investigated the expression of genes encoding specific receptors with that of growth factors in SHPCs and their surrounding MHs. LMD separated SHPC clusters and surrounding MHs in the livers transplanted with BM-MCs, and the expression of genes encoding cytokine receptors was examined via qRT-PCR. As shown in Supplemental Figures 6A–C, the expression of c-*Kit* and *Egfr* was significantly increased in the livers transplanted with BM-MCs. Moreover, BM-MC-transplanted livers tended to express high levels of *IL6r*, although this difference was not significant*.* As shown in Supplemental Figure 6D, *Mki67* expression in MHs was suppressed in both control and BM-MC-transplanted livers in which Ret-treatment had inhibited MH growth. Clearly, although both SCF and IL-6 can equally transduce signals in both MHs and SHPCs, only SHPCs can respond to these signals.

### Administration of EVs derived from BM-MCs transfected with miR-146a-5p to Ret/PH rat livers

**Fig. 9:**
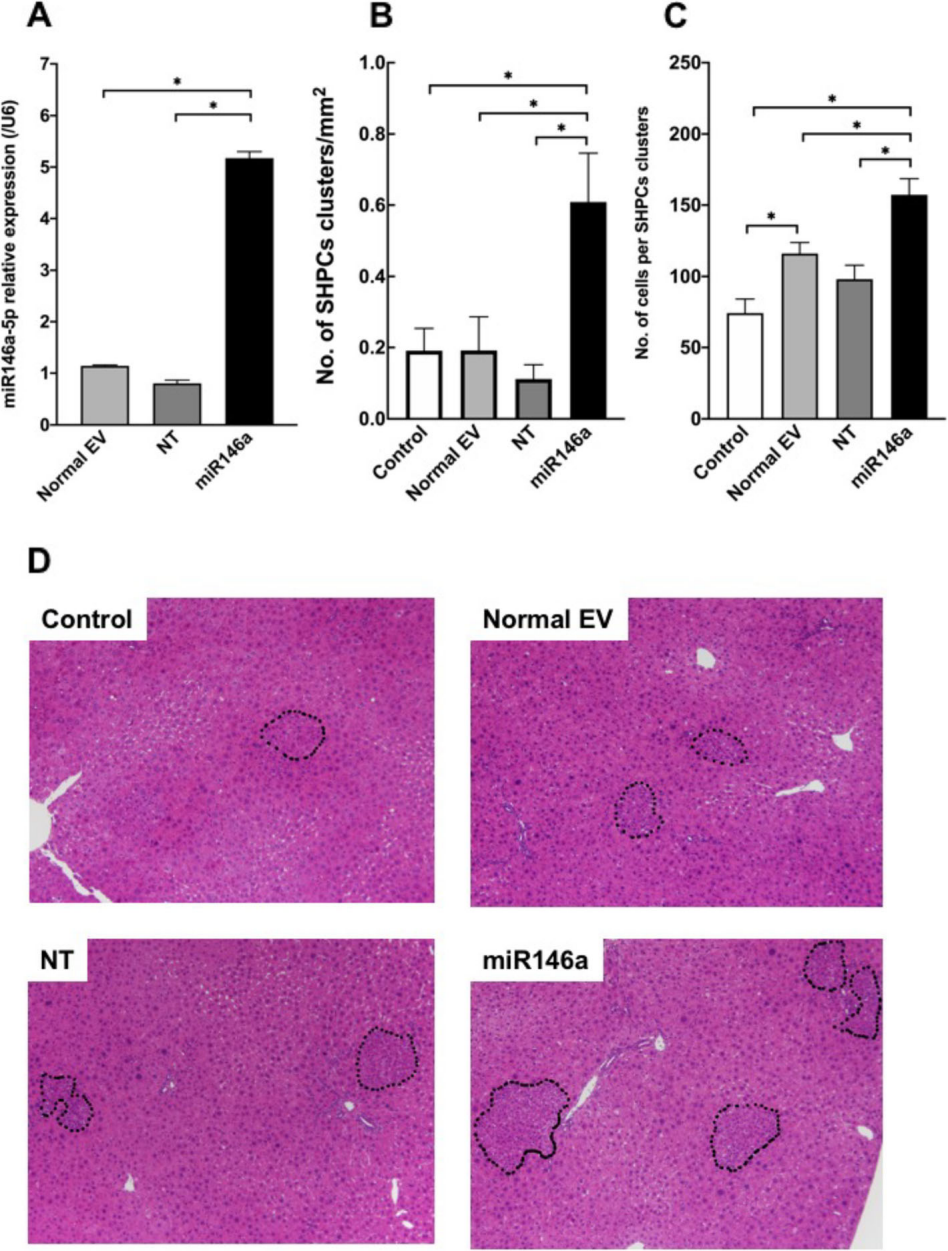
Administration of EVs derived from BM-MCs transfected with *miR-146a-5p* to Ret/PH rat livers. EVs derived from the conditioned medium of cultured BM-MCs transfected with miR-146a-5p for 48 h were administered, and the livers were examined at 14 days post-administration. **a** The amounts of *miR146a-5p* in the EVs secreted by BM-MCs with and without transfection were confirmed using qRT-PCR. Bars show SEs. Asterisks indicate statistically significant differences; *p* < 0.05. **b** The number of SHPC clusters per liver area and **c** the number of cells per cluster is shown. Bars show SEs and asterisks indicate statistically significant differences, *p* < 0.05. **d** Photos of SHPC clusters in the livers of control, EVs (normal EV), EVs secreted by BM-MCs transfected with negative control (NT) or *miR-146a-5p* (miR-146a). The samples were stained with H-E. SHPCs clusters were surrounded by dotted lines: scale bar, 200 μm

We examined the effects of *miR-146a-5p* overexpression in EVs on the growth of SHPCs. Normal EV, NT-EVs (NT), and *miR-146a-5p* overexpressed EVs (miR146a) were administered to Ret/PH-treated rat livers via the spleen. As shown in Fig. 9a, the expression of *miR-146a-5p* was 5 times higher in miR146a than in those from NT. Histological analysis revealed that EVs, including five times larger amounts of miR-146a, induced three times more SHPC clusters than other EVs (Fig. 9b). The size of SHPC clusters in the miR146a was significantly larger than those in control EV and negative-control (NT) (Fig. 9c). As shown in Supplemental Figure 7, the expression of hepatocytic highly differentiated function-related genes in SHPCs with BM-MCs transplantation was not significantly induced. However, the expression of *Cyp2b1* gene was significantly increased in MHs surrounding SHPCs stimulated by EVs with large amounts of *miR-146a-5p*.

## Discussion

This study demonstrated that transplantation with BM-MCs could enhance the regeneration of livers in Ret/PH model rats. Our histological analyses revealed that the increased emergence and expansion of SHPCs contributed to this liver regeneration. As previously reported [18], hepatic Thy1^+^ cells secrete EVs to stimulate the SHPC growth via IL-17RB signaling. Although we observed similar SHPC growth, the effects of BM-MCs were distinct from those of hepatic Thy1^+^ cells. BM-MCs induced a much larger expansion of SHPCs, compared to that induced by Thy1^+^ cells, even though BM-MCs may induce weaker IL-17RB signaling. One reason why this signal is weaker in BM-MCs relative to hepatic Thy1^+^ cells is that the contribution of SECs, mediated by IL-17B secretion, is not apparent in livers transplanted with BM-MCs. The observation that GdCl_3_-mediated inactivation of Kupffer cells in livers transplanted with hepatic Thy1^+^ cells resulted in the suppression of SHPC growth indicates that the phagocytosis of donor cells is essential to the ability of Kupffer cells to secrete IL-25. In livers transplanted with BM-MCs, the observation that some factors secreted by donor cells directly affected SHPCs might be significant. Therefore, it is feasible to expect that the relatively long retention of BM-MCs, which escape phagocytosis by Kupffer cells, may increase the secretion of both EVs and cytokines and the consequent paracrine effects upon neighboring cells.

The EVs secreted by BM-MCs contained miRNAs, of which *miR-146a-5p*, *miR-146b-5p*, *miR-221-3p*, and *miR-222-3p* were more enriched in EVs derived from BM-MCs than in those from Thy1^+^ cells. Of these miRNAs, only *miR-146a-5p* could enhance the growth of SHs in vitro. Although direct *miR-146a-5p* induction in SHPCs has not been studied experimentally, administration of EVs containing larger amounts of miR-146a-5p*miR-146a-5p* could significantly increase about 3 times the number of SHPC clusters than the original EVs, and the size of each cluster was also expanded. These results suggest that this miRNA could stimulate the emergence and the growth of intrinsic hepatic progenitor cells. Previous reports have described several functions of *miR-146a-5p*, including the regulation of immune mechanisms [32–34], hepatic lipid and glucose metabolism [35, 36], hepatocellular carcinoma behaviors [37–40], and cell stemness [41]. This miRNA also acts as a tumor suppressor to reduce the invasion and cancer cell migration of hepatocellular carcinoma [37]. Also, *miR-146a-5p* reduces the expression of *c-met* in colorectal cancer cells to suppress metastasis to the liver [42]. In contrast, *miR-146a-5p* has also been described as an activator of cell proliferation. Mainly, *miR-146a-5p* promoted the growth of human osteosarcoma by targeting the ZNRF3/GSK3β/β-catenin pathway [43] and enhanced the migration and invasion of human colorectal cancer cells by targeting the carboxypeptidase M/src-FAK pathway [44]. Hsieh et al. reported that human bone marrow MSCs expressed *miR-146a-5p*, which regulated the stemness, motility, and proliferation of these cells by targeting CXCL12 and SIKE1 [41].

Studies have demonstrated the involvement of cytokine signaling in the expansion of SHPCs in Ret/PH-treated rat livers [18, 45]. We previously reported that IL-17RB signaling played an essential role in the emergence and expansion of SHPCs [18]. IL-6 signaling was reported as a significant activator of SHPC growth in Ret/PH-treated rat livers [45]. SCF is a well-known hematopoietic factor involved in the maturation and differentiation of multiple types of bone marrow-derived cells [46, 47]. As the liver is a site of early hematopoietic activity, SCF may affect the fetal liver. The adult liver has a large reserve of SCF, and the hepatic SCF levels change dramatically after PH in mice [48]. Furthermore, in vitro and studies have shown that SCF functions as a hepatic mitogen induced by IL-6. In this study, our comprehensive analysis of gene expression and subsequent qRT-PCR confirmation revealed that *Il6r*, *Egfr*, and c-*Kit* significantly increased in the SHPCs of livers transplanted with BM-MCs. Also, the results of our cytokine array demonstrated that EVs derived from BM-MCs were rich in IL-6 and SCF.

Previously, treatment with miR-146a mimics was reported to increase the secretion of IL-6 in BM-MCs [49]. Furthermore, treatment with platelet-derived growth factor stimulated the secretion of EVs in human adipose mesenchymal stem cells, which produce EVs that contain SCF [50]. In the present experiment, we demonstrated that the administration of IL-6 and SCF stimulated the growth of SHs in vitro and that the expression of genes encoding the receptors was similarly upregulated in both MHs and SHPCs from livers transplanted with BM-MCs. Although these cytokines could transduce signals in both MHs and SHPCs, only SHPCs proliferated in the Ret-treated livers transplanted with BM-MCs. The observation that neither SCF nor IL-6 could also enhance the growth of SHs treated with *miR-146a-5p* suggests that this miRNA may play a significant role in expanding SHPCs in livers transplanted with BM-MCs. However, EVs administration reveals that EVs containing *miR-146a-5p* tend to induce the gene expression relating to differentiated hepatic functions such as *C/EBPα, Cyp1a2*, and *Cyp2b1* of more MHs than SHPCs. *miR-146a-5p* may not be involved in the mechanism to induce differentiated hepatic functions such as *Cyp1a2* and *Cyp2b1*.

SHPCs are known to express phenotypic characteristics of fetal hepatoblasts, hepatic oval cells, and MHs [11–17]. Therefore, the origin of SHPCs remains controversial. Chen et al. demonstrated that hepatic oval cells, but not MHs [13], were the origin of SHPCs through experiments in which DPPIV chimeric rat livers were transplanted with MHs, while Best et al. reported that SHPCs were a population of liver progenitor cells distinct from oval cells through experiments using Ret/PH models with 4,4-diaminodiphenylmethane [14]. Avril et al. showed that MHs were the source of SHPCs in Ret/PH model rats in which MHs had been transfected with the gene encoding β-galactosidase [15]. SHPCs were also shown to express low levels of cytochrome P450 enzymes [12]. Low cytochrome P450 activity confers resistance to the mito-inhibitory effects of retrorsine and enables the selective growth of SHPCs to form large clusters. Gordon et al. suggested that SHPCs represent a unique parenchymal progenitor cell population phenotypically distinct from MHs, biliary epithelial cells, and oval cells [12]. We previously reported that the engrafted CD44^+^ SHs (hepatic progenitor cells) in Ret/PH model rat livers were distinct from SHPCs [17]. Although a single SH could proliferate and form a focus in the recipient’s liver, these cells expressed very low levels of C/EBPα and induced very immature sinusoid formation in focus. In contrast, SHPCs expressed relatively high levels of C/EBPα expression, and the sinusoidal networks were well maintained. In this study, the DNA microarray clustering analysis suggested that SHPCs and MHs from the livers transplanted with BM-MCs had similar gene expression patterns. Possibly, MHs, rather than hepatic progenitor cells, are the origin of SHPCs.

## Conclusions

In the present study, we clarified that *miR-146a-5p* contained in the EVs secreted by BM-MCs plays an important role in the emergence and the expansion of SHPCs. Also, the fact that the enhanced expression of *miR-146a-5p* in BM-MCs could increase the number of SHPCs may have a possibility to awake the dormant progenitors in severely damaged livers. This finding may provide a valuable clue to facilitate the development of therapeutics for severe liver diseases based on the administration of EVs or their contents, including microRNAs, rather than transplantation with BM-MCs. Further experiments must be performed to elucidate whether the transduction of *miR-146a-5p* into livers can activate intrinsic hepatocytic progenitor cells and whether unhealthy hepatocytes can be replaced with regenerated hepatocytes.

## Supplementary Information

**Additional file 1: Supplemental figure 1.** Expression of IL17RB signaling-related molecules in livers of Ret/PH models with BM-MCs transplantation. **Supplemental figure 2.** Gene expression analysis of hepatic marker in SHPCs after BMMC transplantation. **Supplemental figure 3.** KEGG pathway analysis of SHPCs between with and without BM-MCs transplantation. **Supplemental figure 4.** Heatmap of a miRNA array analysis of hepatic Thy1^+^ cells and BM-MCs. **Supplemental figure 5.** Heatmap of a cytokine array analysis of hepatic Thy1^+^ cells and BM-MCs. **Supplemental figure 6.** qRT-PCR analysis of cytokine receptor gene expression in SHPCs after BM-MC transplantation. **Supplemental figure 7.** Gene expression analysis of hepatic markers in SHPCs with overexpression of *miR-146a-5p*. **Supporting TABLE S1.** List of antibodies used in the experiments. **Supporting TABLE S2.** List of primers used in the experiments of real-time PCR.

## Data Availability

All DNA microarray data are registered in the GEO database (Accession No. GSE154022).

## Abbreviations

BM-MCs: Bone marrow-derived mesenchymal cells
BrdU: 5-bromo-2′-deoxyuridine
CM: Conditioned medium
DPPIV: Dipeptidylpeptidase IV
EGFR: Epithelial growth factor receptor
EVs: Extracellular vesicles
GalN: d-galactosamine
GdCl_3_: Gadolinium chloride
IL-6: Interleukin-6
IL-17RB: Interleukin-17 receptor B
LI: Labeling index
LMD: Laser microdissection
LSPCs: Liver stem/progenitor cells
MHs: Mature hepatocytes
MSCs: Mesenchymal stem cells
PH: 70% partial hepatectomy
qRT-PCR: Quantitative real-time polymerase chain reaction
Ret: Retrorsine
SCF: Stem cell factor
SECs: Sinusoidal endothelial cells
SHs: Small hepatocytes
SHPCs: Small hepatocyte-like progenitor cells
