# Supplementary material for: Extracellular vesicles containing miR-146a-5p secreted by bone marrow mesenchymal cells activate hepatocytic progenitors in regenerating rat livers
Source: Stem Cell Res Ther. 2021 May 29;12:312. doi: 10.1186/s13287-021-02387-6 (PMC8164814; doi:10.1186/s13287-021-02387-6)
Supplement: Supplementary file 1 — Additional file 1: Supplemental figure 1. Expression of IL17RB signaling-related molecules in livers of Ret/PH models with BM-MCs transplantation. Supplemental figure 2. Gene expression analysis of hepatic marker in SHPCs after BMMC transplantation. Supplemental figure 3. KEGG pathway analysis of SHPCs between with and without BM-MCs transplantation. Supplemental figure 4. Heatmap of a miRNA array analysis of hepatic Thy1+ cells and BM-MCs. Supplemental figure 5. Heatmap of a cytokine array analysis of hepatic Thy1+ cells and BM-MCs. Supplemental figure 6. qRT-PCR analysis of cytokine receptor gene expression in SHPCs after BM-MC transplantation. Supplemental figure 7. Gene expression analysis of hepatic markers in SHPCs with overexpression of miR-146a-5p. Supporting TABLE S1. List of antibodies used in the experiments. Supporting TABLE S2. List of primers used in the experiments of real-time PCR. [file 13287_2021_2387_MOESM1_ESM.docx]

**Supplemental information**

**Supplemental figure 1. Expression of IL17RB signaling-related molecules in livers of Ret/PH models with BM-MCs transplantation.**

Double immunohistochemistry for IL17RB/HNF4α, IL17B/ SE-1 (a marker of SECs), and IL25/CD68 (a marker of Kupffer cells) was performed using Ret/PH model rat livers with (B) and without BM-MCs (A) at 14 days after transplantation. The areas of SHPC clusters are surrounded by white dotted lines. Yellow arrows indicate double-positive cells. Scale bars, 100 μm. All images are at the same magnification.

**
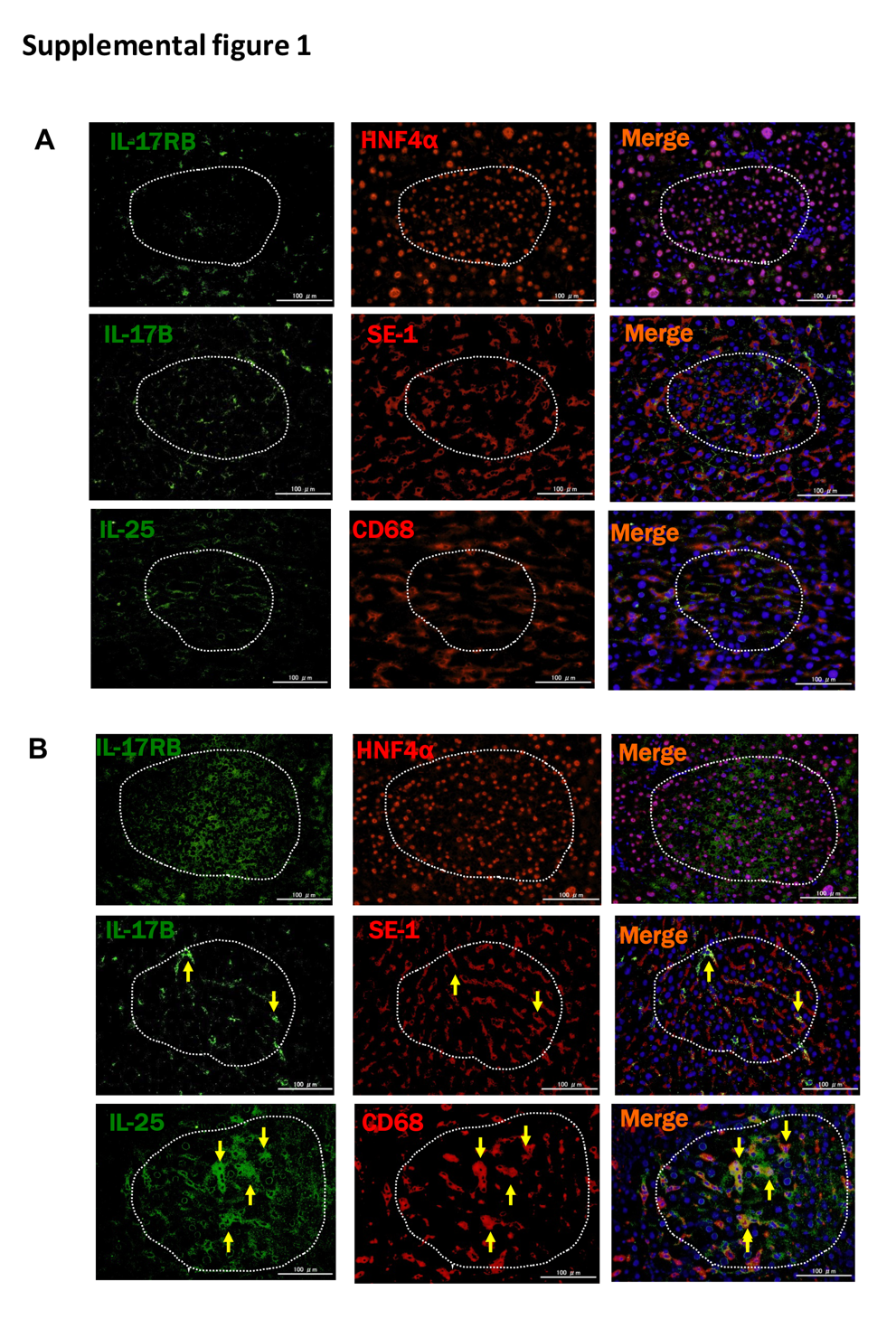
**

**Supplemental figure 2. Gene expression analysis of hepatic marker in SHPCs after BM-MC transplantation.**

(A) The images reflect representative images of enucleated SHPC clusters before (Pre) and after (Post) laser microdissection in livers receiving BM-MCs transplantation. (B) Differences in the expression of genes encoding *Cyp1a2*, *Cyp2b*, *CPS*, *C/EBPα* and *Albumin* in SHPCs and MHs near to SHPCs from livers subjected or not to BM-MC transplantation. Bars are SEs. Asterisks indicate statistically significant differences relative to the Control (*p* < 0.05).

**
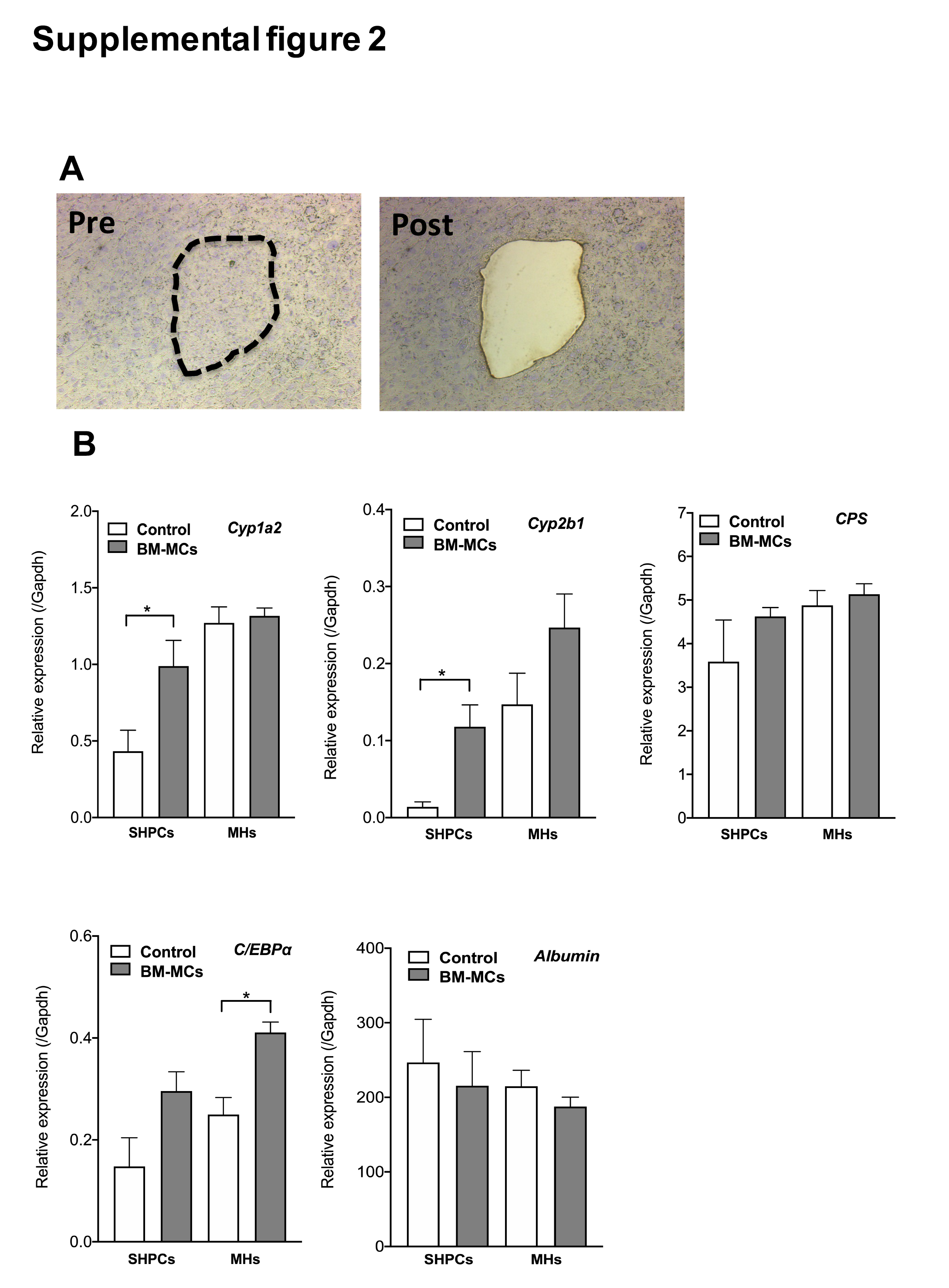
**

**Supplemental figure 3. KEGG pathway analysis of SHPCs between with and without BM-MCs transplantation.**

Gene analysis of SHPCs separated from livers with or without BM-MCs transplantation recovered by laser microdissection (LMD). (A) All gene expressions were analyzed in a volcano plot. The horizontal dotted line indicates that the *P*-value is 0.05 in a Student’s *t*-test. Plots of genes above this dotted line indicate significant differences in the Student’s *t*-test. The X-axis indicates the fold change (FC) in gene expression on a log2 scale. All genes that show significant differences were analyzed in KEGG pathway analysis using Database for Annotation, Visualization and Integrated Discovery (DAVID) v6.7 (<http://david.abcc.ncifcrf.gov>). A total of 275 genes were up-regulated (B), and 75 were down-regulated (C) in SHPCs with BM-MCs transplantation compared with control SHPCs. (D) Heatmap of up-regulated genes included in rno 04010: MAPK signaling pathway. (E) Heatmap of down-regulated genes included in rno 04115: p53 signaling pathway. (F) qRT-PCR analysis of gene expression of MAPK and p53 signaling in SHPCs after BM-MC transplantation. Differences in gene expressions, *Mapk1*, *Jun, p16, p21, p27, and p53*, between SHPCs and MHs separated from livers with or without BM-MC transplantation. Asterisks indicate statistically significant differences relative to the Control (*p* < 0.05).

**
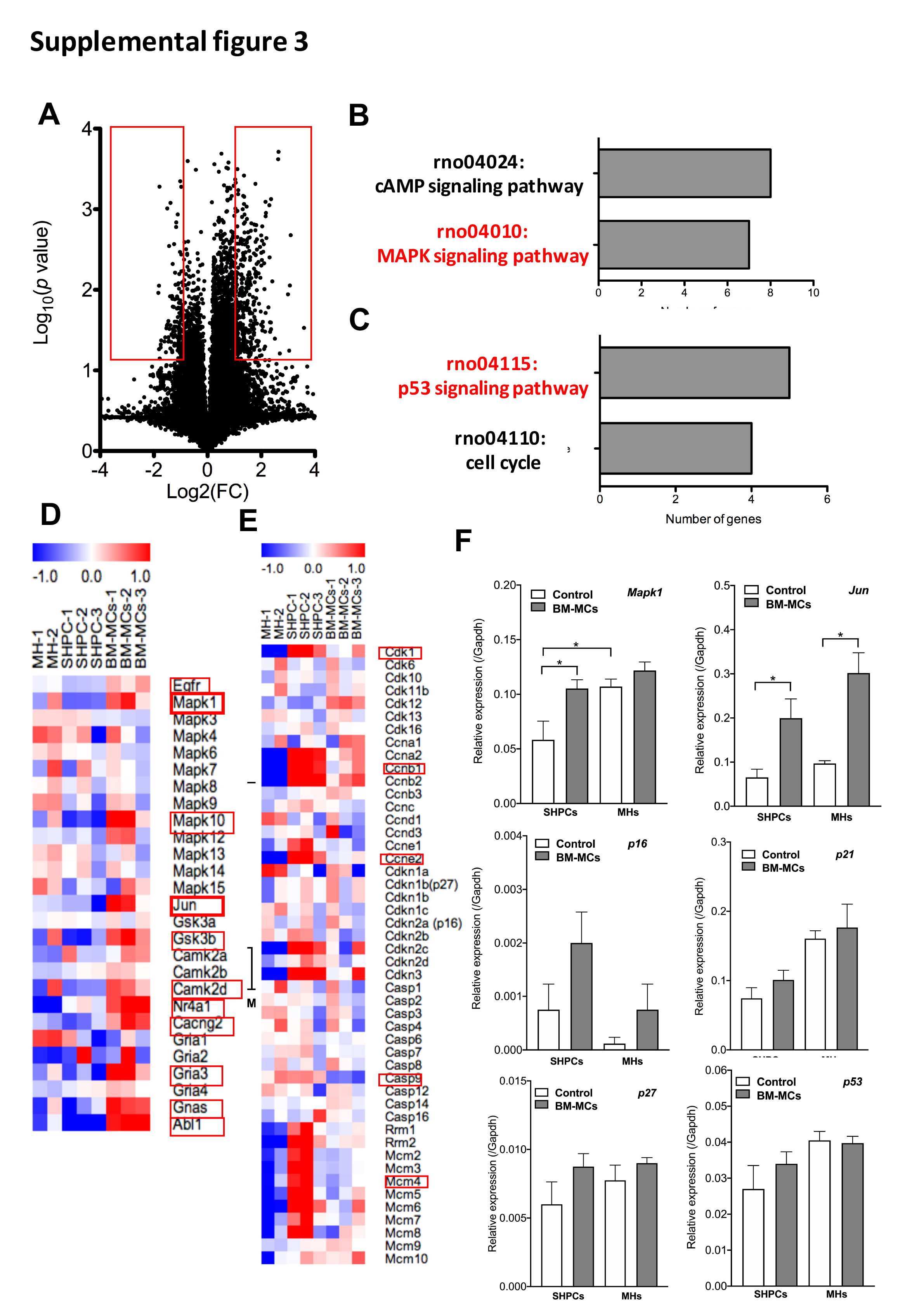
**

**Supplemental figure 4. Heatmap of a miRNA array analysis of hepatic Thy1^+^ cells and BM-MCs.**

**
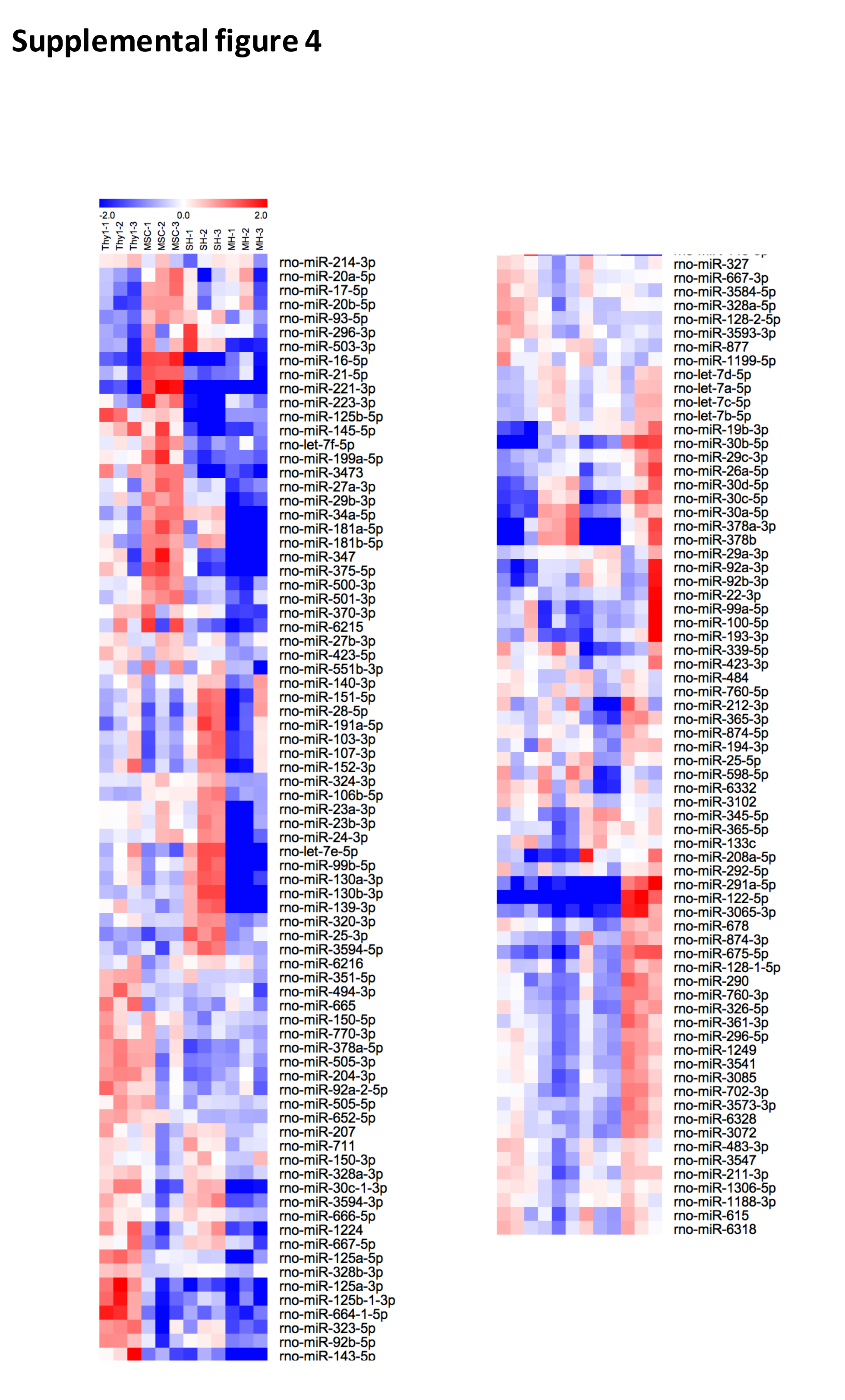
**

**Supplemental figure 5. Heatmap of a cytokine array analysis of hepatic Thy1^+^ cells and BM-MCs.**


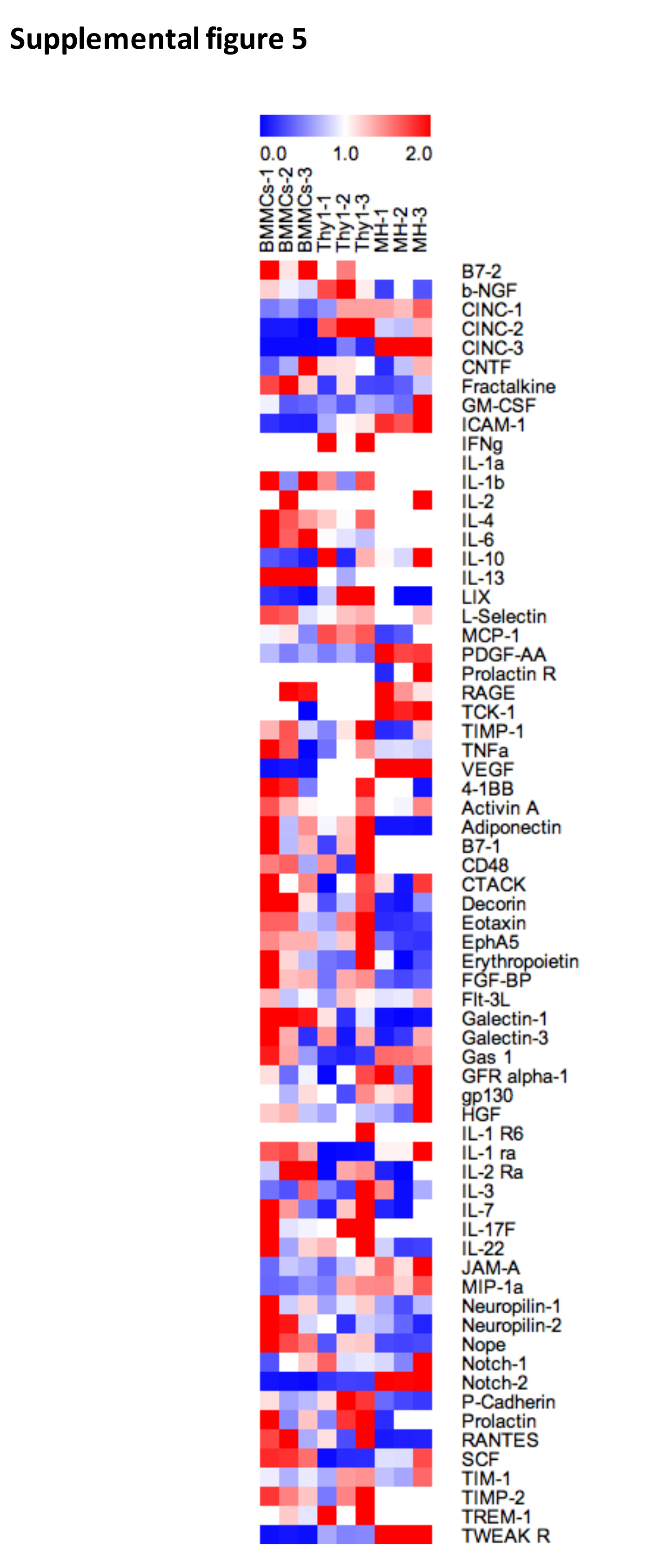


**Supplemental figure 6. qRT-PCR analysis of cytokine receptor gene expression in SHPCs after BM-MC transplantation.**

Differences in the expression of genes encoding SCF (*Kit*), IL-6 (*Il6r*), EGF receptor (*Egfr*), and *Mki67* in SHPCs and MHs near to SHPCs separated from livers with or without BM-MC transplantation. Asterisks indicate statistically significant differences, *p* < 0.05.

**
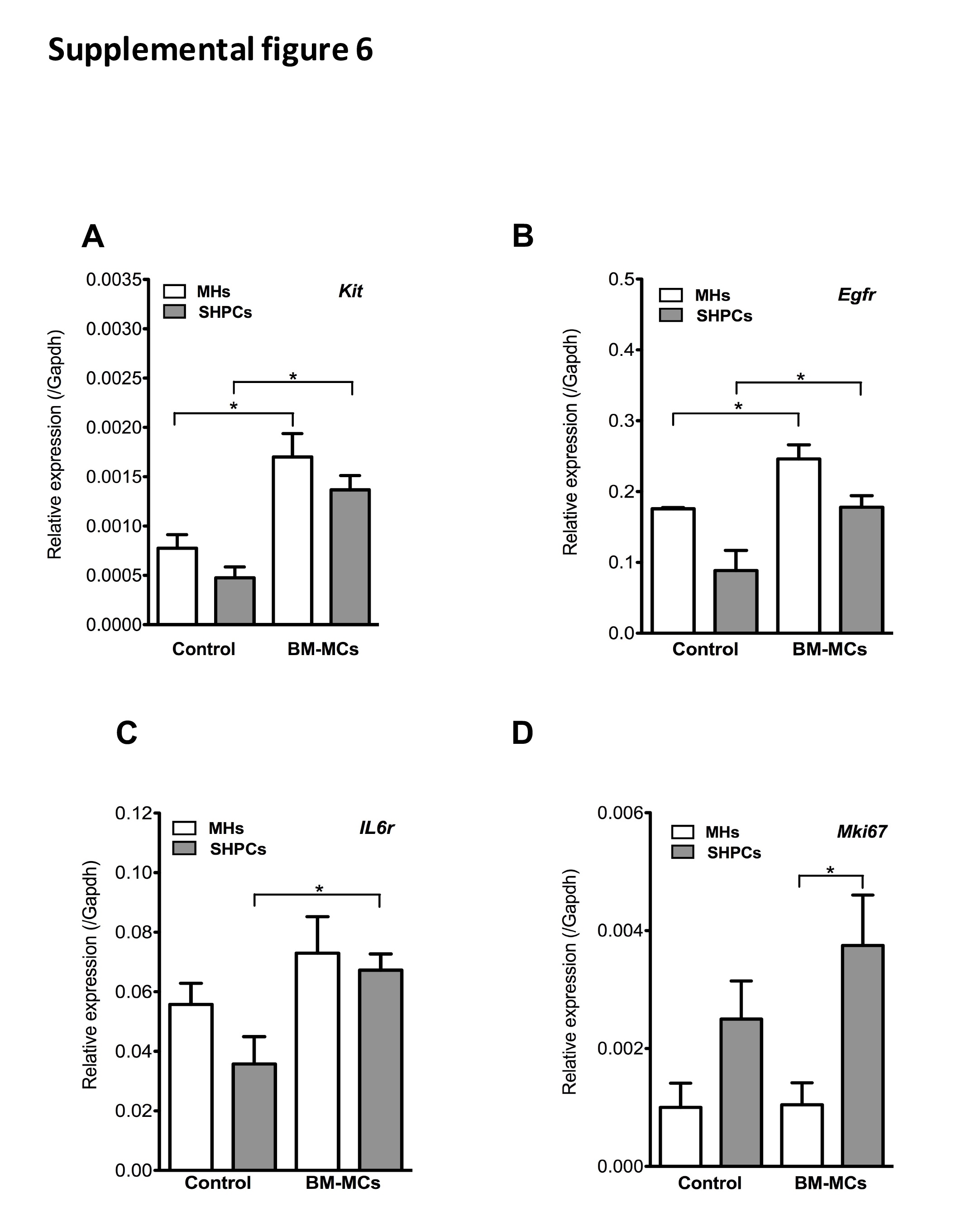
**

**Supplemental figure 7. Gene expression analysis of hepatic markers in SHPCs with overexpression of miR-146a-5p.**

Differences in the expression of genes encoding *Albumin* , *CPS*, *C/EBPα, Cyp1a2*, and *Cyp2b*, in SHPCs and MHs near SHPCs separated from livers with or without BM-MC transplantation. Asterisks indicate statistically significant differences, *p* < 0.05.

**
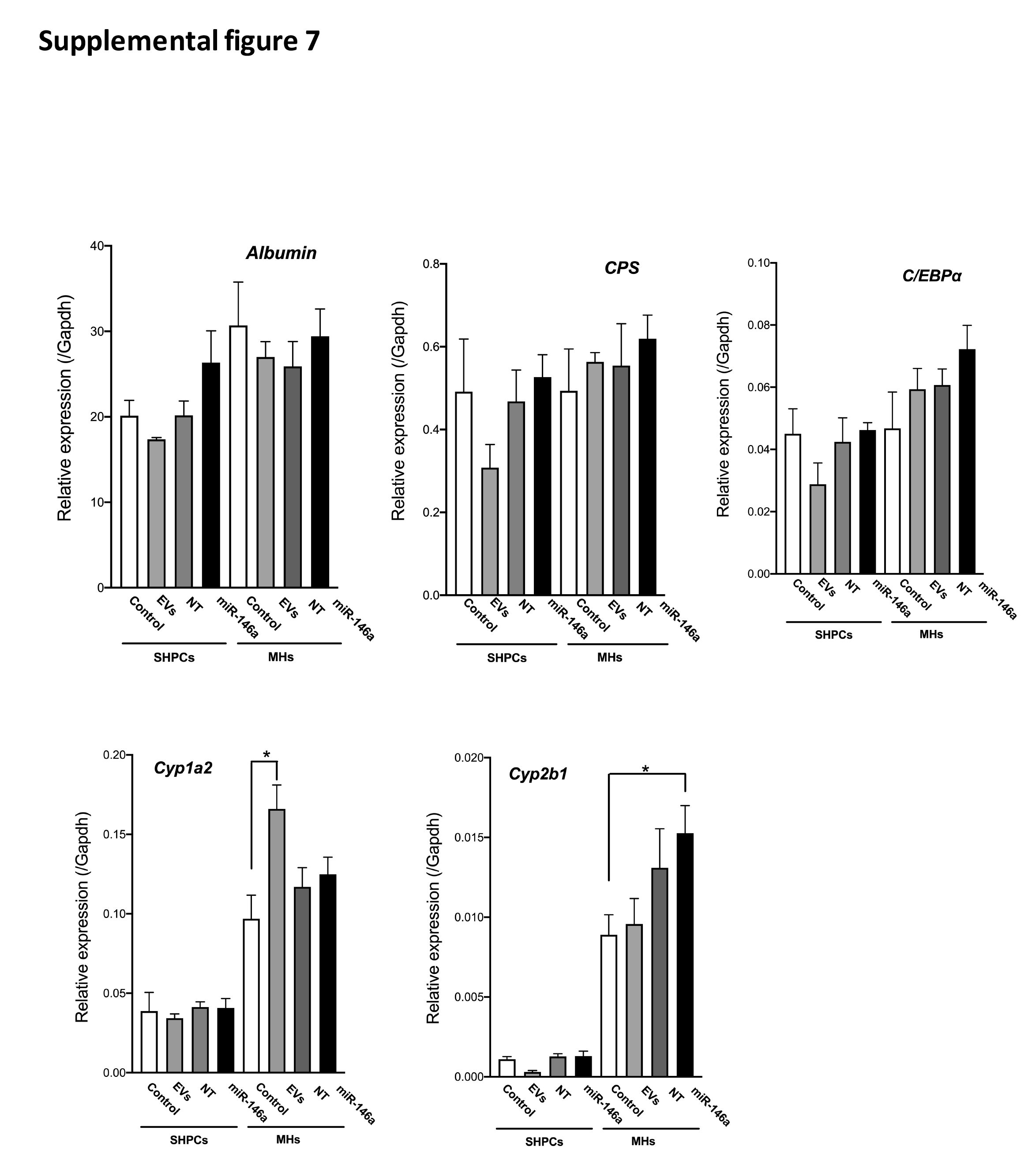
**

**Supporting TABLE S1** List of antibodies used in the experiments.

| Antibodies | Host | Supplier | Dilution |
| --- | --- | --- | --- |
| CD90 (Thy1) | Mouse | Serotec, Raleigh, NC (cat. no. MCA47R) | 1:500 |
| CD73 | Mouse | BD Biosciences Pharmingen, Franklin Lakes, NJ (cat. no. 551123) | 1:200 |
| CD44 | Mouse | BD Biosciences Pharmingen, Franklin Lakes, NJ (cat. no. 554869) | 1:1000 |
| CD29 | hamster | BD Biosciences Pharmingen, Franklin Lakes, NJ (cat. no. 555003) | 1:100 |
| CD34 | mouse | Santa Cruz, Santa Cruz, CA (cat. no. sc-19621) | 1:100 |
| CD11b | rabbit | Novousbio, UK (cat. no. NB110-40766) | 1:100 |
| CD45 | mouse | Serotec, Raleigh, NC (cat. no. MCA43a) | 1:100 |
| CD63 | mouse | Bio-Rad, USA (cat.no. MCA4754T) | 1:500 |
| HSP70 | rabbit | System biocience,Inc. CA (cat.no. EXOAB-sp70A-1) | 1:500 |
| Actin | goat | Santa Cruz, Santa Cruz, CA (cat. no. sc-1616) | 1:500 |
| IL17RB | Rabbit | Bioss, Boston, USA (cat. no. bs-2610R) | 1:500 |
| IL17B | Rabbit | Cloud-Clone Corp, USA (cat. no. PAB700Ra01) | 1:200 |
| IL25 | Goat | Santa Cruz, Santa Cruz, CA (cat. no. sc-22148) | 1:200 |
| Hepatocyte nuclear factor 4α (HNF4α) | Goat | Santa Cruz, Santa Cruz, CA (cat. No. sc-6556) | 1:200 |
| SE-1 | Mouse | Immuno-Biological Lab., Takasaki, Japan(cat. no. 10078) | 1:200 |
| CD68 | Mouse | Serotec, Raleigh, NC (cat. no. MCA341R) | 1:500 |
| BrdU | Mouse | DakoCytomation, Glostrup, Denmark (cat. no. M0744) | 1:200 |
| Anti-mouse IgG HRP conjugate | Goat | Bio-Rad, USA (cat.no. 170-6516) | 1:10000 |
| Anti-rabbit IgG HRP conjugate | Goat | Bio-Rad, USA (cat.no. 170-6515) | 1:10000 |
| Anti-goat IgG HRP conjugate | Rabbit | Bio-Rad, USA (cat.no. 172-1034) | 1:10000 |
| Mouse IgG2a+b microbeads | Rat | Miltenyi Biotec, Bergisch Gladbach, Germany (cat. no. 130-047-201) | 1:5 |
| Alexa 488-conjugated anti-mouse | Goat | Molecular Probes, Eugene, OR (cat. no. A11029) | 1:500 |
| Alexa 488-conjugated anti-mouse | Rabbit | Molecular Probes, Eugene, OR (cat. no. A11059) | 1:500 |
| Alexa 488-conjugated anti-hamster | Goat | Molecular Probes, Eugene, OR (cat. no. A21110) | 1:500 |
| Alexa 488-conjugated anti-rabbit | Donkey | Molecular Probes, Eugene, OR (cat. no. A21206) | 1:500 |
| Alexa 594-conjugated anti-mouse | Goat | Molecular Probes, Eugene, OR (cat. no. A11005) | 1:500 |
| Alexa 594-conjugated anti-rabbit | Donkey | Molecular Probes, Eugene, OR (cat. no. A21207) | 1:500 |
| Alexa 594-conjugated anti-goat | Donkey | Molecular Probes, Eugene, OR (cat. no. A11058) | 1:500 |
| Biotin-conjugated anti-mouse | Horse | Vector Laboratories, Burlingame, CA (cat. no. BA-2000) | 1:200 |

**Supporting TABLE S2** List of primers used in the experiments of real-time PCR.

| Genes | Real-Time PCR Primers(Assay ID) |
| --- | --- |
| Mki67 | Rn01451446_m1 |
| Mapk1 | Rn00671828_m1 |
| Jun | Rn99999045_m1 |
| p16(Cdkn2a) | Rn00580664_m1 |
| p21(Cdkn1a) | Rn01427989_m1 |
| p27(Cdkn1b) | Rn00582195_m1 |
| p53(Tp53) | Rn00755717_m1 |
| Casp3 | Rn00563902_m1 |
| Casp9 | Rn00573942_m1 |
| Kit | Rn00581212_m1 |
| IL6r | Rn01495381_m1 |
| EGFr | Rn00561225_m1 |
| Gapdh | Rn01775763_g1 |
| miR-144a-3p | 002676 |
| miR-146a-5p | 000468 |
| miR-146b-5p | 002755 |
| miR-221-3p | 000524 |
| miR-222-3p | 002276 |
| U6 | 001973 |
|  |  |
